# Ex vivo MRI atlas of the human medial temporal lobe: characterizing neurodegeneration due to tau pathology

**DOI:** 10.1186/s40478-021-01275-7

**Published:** 2021-10-24

**Authors:** Sadhana Ravikumar, Laura E. M. Wisse, Sydney Lim, Ranjit Ittyerah, Long Xie, Madigan L. Bedard, Sandhitsu R. Das, Edward B. Lee, M. Dylan Tisdall, Karthik Prabhakaran, Jacqueline Lane, John A. Detre, Gabor Mizsei, John Q. Trojanowski, John L. Robinson, Theresa Schuck, Murray Grossman, Emilio Artacho-Pérula, Maria Mercedes Iñiguez de Onzoño Martin, María del Mar Arroyo Jiménez, Monica Muñoz, Francisco Javier Molina Romero, Maria del Pilar Marcos Rabal, Sandra Cebada Sánchez, José Carlos Delgado González, Carlos de la Rosa Prieto, Marta Córcoles Parada, David J. Irwin, David A. Wolk, Ricardo Insausti, Paul A. Yushkevich

**Affiliations:** 1 Department of Bioengineering, University of Pennsylvania, Richards Building 6th Floor, Suite D, 3700 Hamilton Walk, Philadelphia, PA 19104 USA; 2 Department of Radiology, University of Pennsylvania, Philadelphia, PA 19104 USA; 3 Department of Neurology, University of Pennsylvania, Philadelphia, PA 19104 USA; 4 Department of Diagnostic Radiology, Lund University, 22242 Lund, Sweden; 5 Department of Pathology, University of Pennsylvania, Philadelphia, PA 19104 USA; 6 Human Neuroanatomy Laboratory, CSIC Neuromax Associated Unit, University of Castilla La Mancha, 02008 Albacete, Spain

**Keywords:** Alzheimer’s disease, Neurofibrillary tangles, Ex vivo MRI, Neurodegeneration, Biomarkers, Co-morbidities

## Abstract

Tau neurofibrillary tangle (NFT) pathology in the medial temporal lobe (MTL) is closely linked to neurodegeneration, and is the early pathological change associated with Alzheimer’s disease (AD). To elucidate patterns of structural change in the MTL specifically associated with tau pathology, we compared high-resolution ex vivo MRI scans of human postmortem MTL specimens with histology-based pathological assessments of the MTL. MTL specimens were obtained from twenty-nine brain donors, including patients with AD, other dementias, and individuals with no known history of neurological disease. Ex vivo MRI scans were combined using a customized groupwise diffeomorphic registration approach to construct a 3D probabilistic atlas that captures the anatomical variability of the MTL. Using serial histology imaging in eleven specimens, we labelled the MTL subregions in the atlas based on cytoarchitecture. Leveraging the atlas and neuropathological ratings of tau and TAR DNA-binding protein 43 (TDP-43) pathology severity, morphometric analysis was performed to correlate regional MTL thickness with the severity of tau pathology, after correcting for age and TDP-43 pathology. We found significant correlations between tau pathology and thickness in the entorhinal cortex (ERC) and stratum radiatum lacunosum moleculare (SRLM). When focusing on cases with low levels of TDP-43 pathology, we found strong associations between tau pathology and thickness in the ERC, SRLM and the subiculum/cornu ammonis 1 (CA1) subfields of the hippocampus, consistent with early Braak stages.

**Supplementary Information:**

The online version contains supplementary material available at 10.1186/s40478-021-01275-7.

## Introduction

Alzheimer’s disease (AD) pathology is characterized by phosphorylated-tau in the form of neurofibrillary tangles (NFTs) and extra-cellular deposits of amyloid-beta (Aβ) [1–3], which are thought to lead to neurodegeneration and cognitive decline. Measures of Aβ, tau and neurodegeneration (A/T/N) can be obtained using positron emission tomography (PET), cerebrospinal fluid (CSF) or structural magnetic resonance imaging (MRI), and are now being used to biologically define AD within a research framework [4]. Compared to Aβ, the accumulation of NFTs in particular is strongly correlated with neurodegeneration and cognitive decline [5–8]. The medial temporal lobe (MTL), which consists of the hippocampal formation and the adjacent entorhinal, temporopolar, perirhinal and parahippocampal cortices, is the earliest cortical region affected by NFT pathology [1, 2]. According to the staging model of Braak and Braak [1, 2], NFTs initially manifest in a specific region of the MTL surrounding the border between the lateral part of the entorhinal cortex (ERC) and transentorhinal cortex. The transentorhinal cortex corresponds to Brodmann area (BA) 35, itself encompassing the medial portion of the perirhinal cortex (PRC). The NFTs then spread further into the ERC before emerging in the subiculum (SUB) and cornu ammonis 1 (CA1) subfield of the hippocampus. Studies have shown that the stratum radiatum lacunosum moleculare (SRLM) layer of SUB/CA is also an early target of NFT pathology [9, 10]. Eventually, the NFTs spread to other cortical regions of the brain. Because of the MTL’s early involvement, its subregions are expected to be among the most sensitive brain regions to early pathological changes in AD.

A growing number of studies have used in vivo structural MRI to derive measurements of subtle volumetric changes in the MTL caused by neurodegeneration [11, 12]. However, the specificity of these measures to AD pathology is limited by the fact that the MTL is also affected by several co-occurring pathologies such as TAR DNA-binding protein 43 (TDP-43), α-synuclein, vascular disease, and even aging [6, 13, 14]. Although the MTL is thought to display different patterns of regional vulnerability to the different pathologies, the specific contribution of NFT pathology to atrophy is not well established [6]. Identifying macroscopic patterns of structural change in the MTL that are specific to underlying NFTs could help guide the development of in vivo MRI biomarkers that are more sensitive to AD-related changes. Several studies have looked at the association between MTL structure and either pathological [15–17], CSF [18–20] or more recently, PET [21–23] measures of tau burden. Although tau PET enables in vivo quantification and 3D mapping of tau burden, it lacks the spatial resolution to obtain measurements within specific MTL subregions affected during the early stages of AD [24]. Most recently, Wisse et al. conducted a postmortem study which investigated the association between the thickness of MTL subregions in ex vivo MRI and semi-quantitative ratings of different neurodegenerative pathologies derived from histology samples taken from the contralateral hemisphere [25]. However, thickness was only sampled at specific hand-picked anatomical locations in the MTL, thus providing only a limited window into the structure/pathology relationships.

Here we develop a methodological framework that allows detailed 3D mapping of the relationships between MTL thickness and pathological measures. We generate the first-of-its-kind high-resolution 3D computational atlas of the human MTL generated by applying groupwise image registration to 200 × 200 × 200 µm^3^ resolution post-mortem MRI scans of intact MTL specimens from twenty-nine brain donors. To overcome challenges due to the complex geometry of the hippocampus and MTL cortex and significant anatomical variability across specimens, we leverage advanced computational anatomy techniques to co-register the individual ex vivo MRI scans and capture the “average” shape of the MTL. Anatomical regions in the atlas are derived by combining information from eleven specimens in which serial Nissl histology was used to annotate MTL and hippocampal subregions based on cytoarchitecture. Leveraging this atlas, we report regional patterns of association between MTL thickness and NFT pathology (measured both in ipsilateral and contralateral hemispheres), while accounting for the confounds of TDP-43 pathology.

## Methods

### Specimen preparation and MRI

Brain hemispheres were obtained from twenty-nine donors; one specimen from the brain bank operated by the National Disease Research Interchange (NDRI), sixteen specimens from autopsies performed at the University of Pennsylvania Center for Neurodegenerative Disease Research (CNDR) and twelve specimens from the University of Castilla-La Mancha (UCLM) Human Neuroanatomy Laboratory (HNL) in Spain. Human brain specimens were obtained in accordance with the University of Pennsylvania Institutional Review Board guidelines, and the Ethical Committee of UCLM. Where possible, pre-consent during life and, in all cases, next-of-kin consent at death was given. CNDR hemispheres were fixed in 10% formalin solution for at least 30 days before extracting intact MTL blocks. HNL cases were fixed by perfusion with 4% paraformaldehyde through both carotid arteries. The blocks were then imaged on a Varian 9.4 T animal scanner at a 200 × 200 × 200 µm^3^ resolution. Details of the imaging protocol are provided in Additional file 1: Section 1.1 and Additional file 1: Fig. S7.

### Histological processing and immunohistochemistry

Following MRI scanning, each of the specimens underwent histological processing for neuropathological examination and neuroanatomical analysis. In twenty-seven specimens, dense serial histology was performed. The specimens were cut into 2 cm blocks using custom molds that were 3D printed to fit each MTL specimen, cryoprotected and sectioned at 50 μm intervals in a sliding microtome coupled to a freezing unit (Microm, Heidelberg). For neuropathological diagnosis, we were interested in examining the anterior ERC at the mid-level of the amygdala, and the dentate gyrus (DG) and CA subfields at the mid-level of the body of the hippocampus. Therefore, for the remaining two specimens that did not undergo serial histology, two tissue blocks were cut at the level of the amygdala and hippocampal body, cryoprotected and sectioned into 50 μm sections. For each of the specimens, two adjacent sections were sampled at the mid-level of the amygdala and the hippocampal body for immunohistochemistry. In each case, the two sections were immunostained for tau and TDP-43 using anti-human PHF-Tau (monoclonal antibody (mAb), mouse, Thermo Scientific, Product Number MN1020, 1:500) and 66318-1-Ig Anti-phospho (409/410) TDP-43 (mAb, mouse, Proteintech, 1:350) respectively. Sections were then mounted on 7.5 cm × 5 cm slides and digitally scanned at 20× resolution.

### Semi-quantitative neuropathology ratings

Regional thickness analysis was performed using histopathology ratings of tau and TDP-43 pathologies derived from the MTL both ipsilateral and contralateral to the one that was scanned. Semi-quantitative ratings for tau and TDP-43 pathology were obtained for the contralateral hemisphere by obtaining tissue samples at the time of autopsy and are available for a subset of twenty-eight donors (CNDR and HNL specimens) in three MTL regions routinely examined in the CNDR neuropathology evaluations [26]. More specifically, the contralateral tissue was embedded in paraffin blocks and cut into 6 μm sections for immunohistochemistry using phosphorylated tau PHF-1 (mAb, 1:1000, a gift from Dr. Peter Davies) to detect phosphorylated tau deposits and pS409/410 (mAb, 1:500, a gift from Dr. Manuela Neumann and Dr. E. Kremmer) to detect phosphorylated TDP-43 deposits. Three MTL locations, namely the ERC, DG and CA, were each visually assigned a semiquantitative rating on a scale of 0–3 i.e. “none (0)”, “rare (0.5)”, “mild (1)”, “moderate (2)” or “severe (3)” [3]. For the analysis, the average rating across all three locations was used as a measure of general pathology burden in a specimen.

To generate the ipsilateral ratings, an expert neuropathologist (D.J.I) provided semi-quantitative ratings of the severity of tau and TDP-43 pathology burden in the MTL using the scanned digital immunohistochemistry images for each of the neurodegenerative pathologies. Once again, the average ipsilateral rating across the three MTL locations was used in the analysis. We note that unlike the contralateral ERC ratings which are based on tissue sampled at the level of the lateral geniculate nucleus, ipsilateral ERC ratings are obtained using anterior ERC sections at the level of the amygdala.

### Manual segmentation of the medial temporal lobe cortex to guide atlas construction

The atlas construction pipeline used in this work relies on segmentations of the MTL and SRLM to guide the registration process. To reduce the manual effort needed to generate these 3D segmentations, we adopted semi-automatic segmentation methods to label the MTL and SRLM [27]. In some specimens, opposing banks of the collateral sulcus can appear fused together due to tight folding patterns and collapsing of cerebrospinal fluid spaces. Therefore, sulcus delineation was explicitly enforced by using different labels to segment the medial and lateral portions of the collateral sulcus. Another challenge in ex vivo datasets is the presence of imaging artifacts caused by small air bubbles trapped in brain folds or tearing of the tissue during the extraction and cutting process. While segmentations of these affected regions can be used to guide the registration, incorporating intensity information from these regions would introduce errors in any intensity-based registration steps. We therefore introduced a separate label to segment regions of the MTL which should be excluded from intensity-based registration, and the eventual thickness analysis. For more details on the semi-automated segmentation approach and segmentation protocol see Additional file 1: Section 1.1 and Additional file 1: Fig. S1.

### MRI atlas generation

An ex vivo MRI atlas of the MTL was generated by leveraging the registration pipeline developed by Adler et al. to build an ex vivo atlas of the human hippocampus [28]. Conventional deformable registration techniques applied to high-resolution ex vivo scans result in poor alignment between specimens. To address this, Adler et al. propose a three-stage algorithm which incorporates segmentations of the structure of interest to initialize groupwise deformable registration. The first two stages perform shape-based alignment using the MTL and SRLM segmentations. This establishes geometric correspondences between specimens and yields an average MTL and SRLM shape. The final stage uses these correspondences to initialize groupwise MRI intensity registration which resolves residual misalignments between specimens. Extending this framework to include the extrahippocampal regions required optimization of the registration metrics and parameters (Additional file 1: Section 1.3 and Additional file 1: Table S1). To better capture the collateral sulcus fold, registrations between medial and lateral banks of the sulcus were optimized separately using the multi-label segmentations. We also introduced the capability to handle image artifacts by masking out affected regions from intensity-based registration computations. The final probabilistic atlas consists of a template (synthetic 3D image capturing the “average” MTL anatomy), a template segmentation, and a set of non-linear diffeomorphic transformations between the template and each individual specimen’s scan.

### Histology reconstruction and cytoarchitectural annotation of MTL subregions

In eleven of the specimens that underwent serial histological processing, every tenth section (i.e., 0.5 mm intervals) in each tissue block was stained for Nissl using 0.25% thionin and digitally scanned at 20× resolution. For each block, the scanned sections were reconstructed into a 3D volume and aligned to MRI space using a custom deformable 3D registration pipeline. Further details of the histology protocol and the approach for 3D reconstruction and matching of histology to MRI are provided in Yushkevich et al. [29].

For each of the specimens, the boundaries between MTL subregions cornu ammonis (CA) 1, CA2, CA3, DG, SUB, presubiculum, parasubiculum, hippocampal amygdala transition area (HATA), SRLM, ERC, BA35, BA36, area TE and the parahippocampal cortex (areas TF and TH) were identified on the basis of cytoarchitectural features in the Nissl stained sections following the anatomical rules presented in the Atlas of the Human Brain by Mai et al. [30]. Annotations were performed on each histology slice by the team of neuroanatomists at UCLM (the hippocampal subfields and ERC were annotated by M.M.A, E.A.P, M.M.R, M.M.L, C.R.P, S.C.S, J.C.D, M.C.P and F.M.R., supervised by R.In; R.In annotated the boundaries of the temporopolar cortex, PRC and PHC, and revised the annotations of the group). The neuroanatomists viewed scanned Nissl slides in an open-source web-based system (https://github.com/pyushkevich/histoannot) and used line drawings and text labels to annotate boundaries between adjacent anatomical regions. Following histology reconstruction and registration to MRI, the boundary annotations were overlaid on the co-registered MRI and histology images. Additionally, the manual segmentations of the whole MTL cortex and SRLM that were used to guide groupwise registration were registered to the histology images to inform the outer MTL boundary. The MRI, histology and MTL segmentation images were displayed side-by-side in ITK-SNAP [31] to facilitate manual tracing of the subfield segmentations in 3D MRI space, which was performed by S.L. with supervision from L.E.M.W and R.In (Additional file 1: Fig. S9).

For each of the eleven specimens, the completed segmentations were then mapped to the MRI atlas using the deformable transformations generated by the groupwise registration pipeline. Note that for each specimen, small gaps in the segmentation may exist between blocks. A consensus segmentation of the MRI atlas was obtained by application of voxel-wise majority voting among the eleven segmentations with slight regularization by a Markov Random Field prior. More details on this approach are provided in the supplemental information, Section S1.4.5 of Adler et al. [28].

### Statistical analysis

Regional thickness of the MTL cortex, hippocampal gray matter, and SRLM was estimated by warping the atlas to the native MRI space of each specimen and performing Voronoi skeletonization (Additional file 1: Section 1.4). To test the effects of tau pathology on regional thickness, we fit a general linear model (GLM) at each vertex on both the MTL and SRLM surfaces with the average rating of tau pathology as the independent variable, thickness as the dependent variable, and age and TDP-43 rating as covariates. More details on the statistical analysis are provided in Additional file 1: Section 1.5.

## Results

### Demographics

**Fig. 1:**
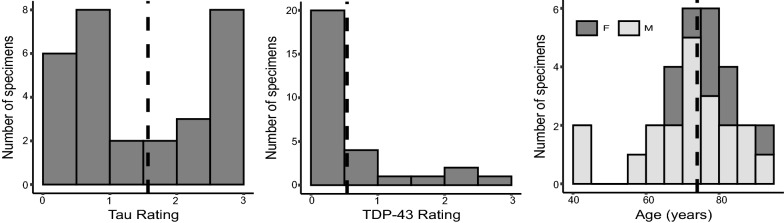
Demographic and diagnostic summaries for the twenty-nine brain donors. The tau and TDP-43 pathology ratings refer to the average rating computed from measurements sampled at three medial temporal lobe locations (entorhinal cortex at the mid-level of the amygdala and subiculum/cornu ammonis and dentate gyrus at the mid-level of the hippocampus). Dashed lines are used to indicate the mean value across specimens

The specimens included in this study contain varying neuropathological diagnoses, including AD neuropathologic change and neuropathological diagnoses such as argyrophilic grain disease (AGD), frontotemporal lobar degeneration (FTLD) with TDP-43 inclusions, cerebrovascular disease and Lewy body disease. Figure 1 summarizes the demographic and neuropathology data for this brain donor cohort. The average ipsilateral tau and TDP-43 ratings across the twenty-nine specimens are 1.50 ± 0.97 and 0.54 ± 0.82 respectively. The average age is 74 years (range 44–93 years). Additional file 1: Table S2 provides more detailed demographic data and pathology information for each specimen.

### Computational atlas of the medial temporal lobe

**Fig. 2:**
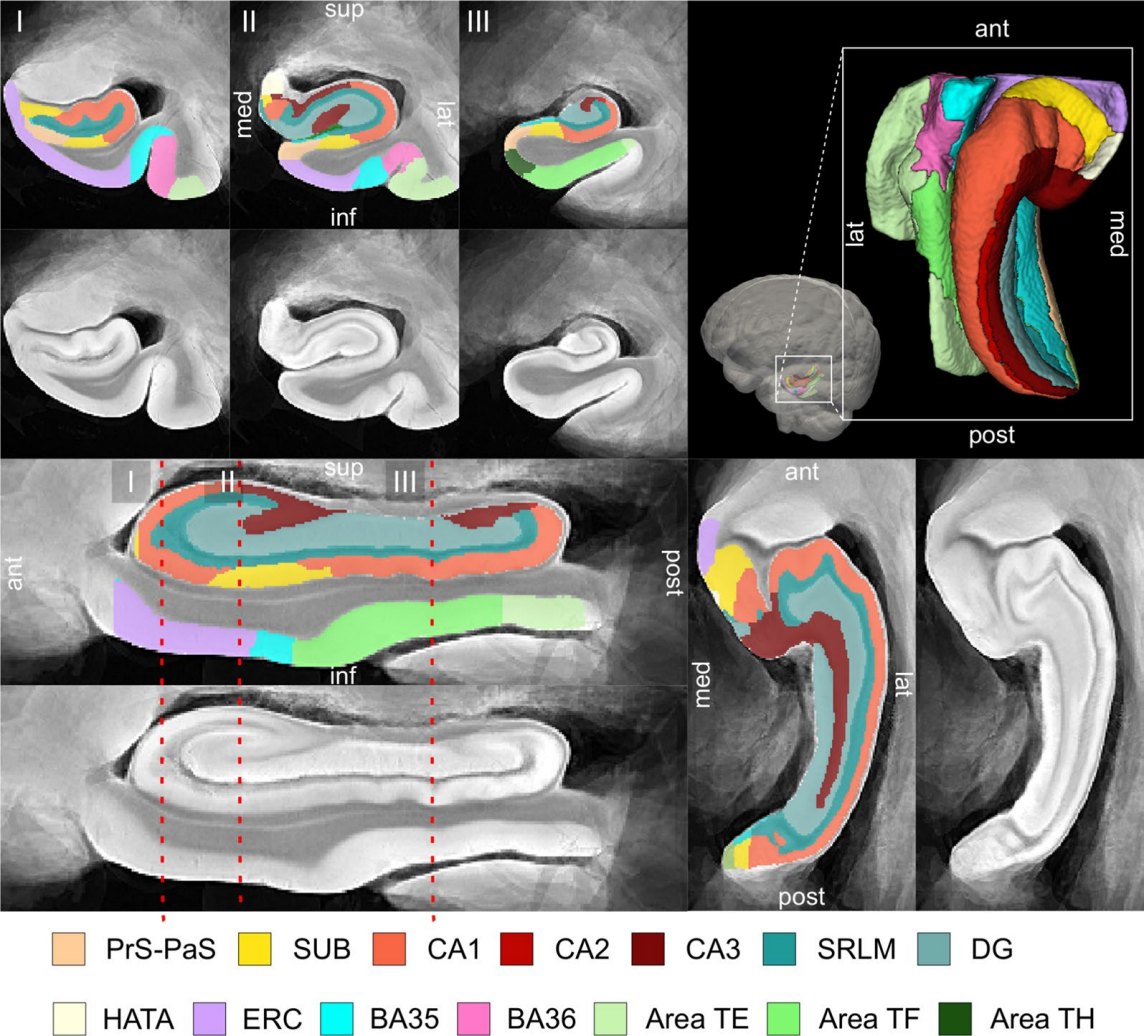
Computational atlas of the medial temporal lobe (MTL) constructed by groupwise registration of the magnetic resonance image (MRI) scans of twenty-nine ex vivo specimens. Three coronal sections are shown ordered from anterior (ant) to posterior (post), indicated as I, II and III, as well as a sagittal and axial section through the MTL. For each section, the “average” MRI is shown with and without the consensus MTL subregion segmentation derived from serial histology in eleven specimens. In the top right, a 3D reconstruction of the MTL atlas is shown along with a 3D brain rendering indicating the location of the MTL within the brain. (*med* medial, *lat* lateral, *sup* superior, *inf* inferior, *SUB* subiculum, *SRLM* stratum radiatum lacunosum moleculare, *CA* cornu ammonis, *DG* dentate gyrus, *HATA* hippocampal amygdala transition area, *ERC* entorhinal cortex, *BA* Brodmann Area)

**Fig. 3:**
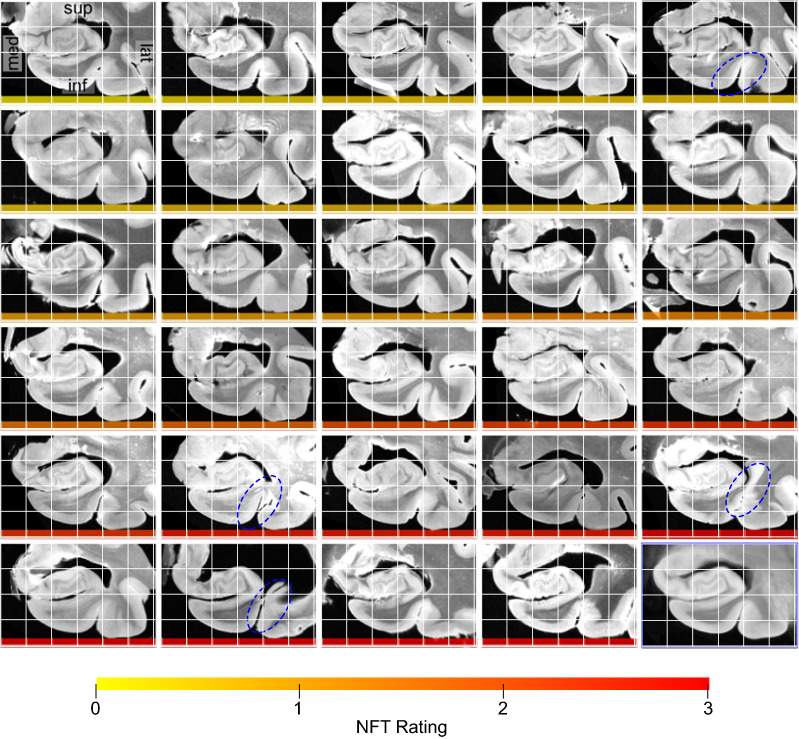
Coronal view of the MRI scans of each of the twenty-nine specimens warped into the space of the MRI atlas. The corresponding atlas image is outlined in blue, in the bottom-right corner. The more similar the warped images are, the better the atlas quality. The dashed blue circles point out examples where the perirhinal cortex (region surrounding the collateral sulcus) was particularly challenging to register due to significant variability in cortical folding patterns. The color bar at the bottom of each image indicates the average neurofibrillary tangle (NFT) rating for that specimen. Yellow represents a rating of 0 (no/rare pathology) and red represents a rating of 3 (severe Alzheimer’s disease)

Figure 2 shows the MRI atlas of the MTL constructed from twenty-nine ex vivo specimens as a synthetic “average” MR image and a consensus MTL subregion segmentation derived from serial histology in eleven specimens. The atlas construction pipeline achieves excellent groupwise alignment between ex vivo MRI scans and captures the average shape of the MTL (Additional file 1: Section 2.1, Additional file 1: Fig. S2, Additional file 1: Fig. S3 and Additional file 1: Fig. S4). Following groupwise registration, each specimen has a pointwise spatial correspondence to this atlas. This correspondence is limited to the region of the MTL cortex which was semi-automatically segmented in each specimen. Figure 3 provides a visualization of the quality of the registration between individual specimen images and the final atlas. In some specimens, the PRC, which includes BA35, was particularly challenging to register due to significant anatomical variability in cortical folding and branching patterns [32, 33]. Overall, the warped specimens look similar to each other following registration, although some minor mis-registrations remain. Quantitative and visual evaluation of atlas quality at different stages of atlas construction, and comparisons with an alternative atlas-building strategy are presented in Additional file 1: Section 2.1.

### Effects of tau pathology on regional thickness

**Fig. 4:**
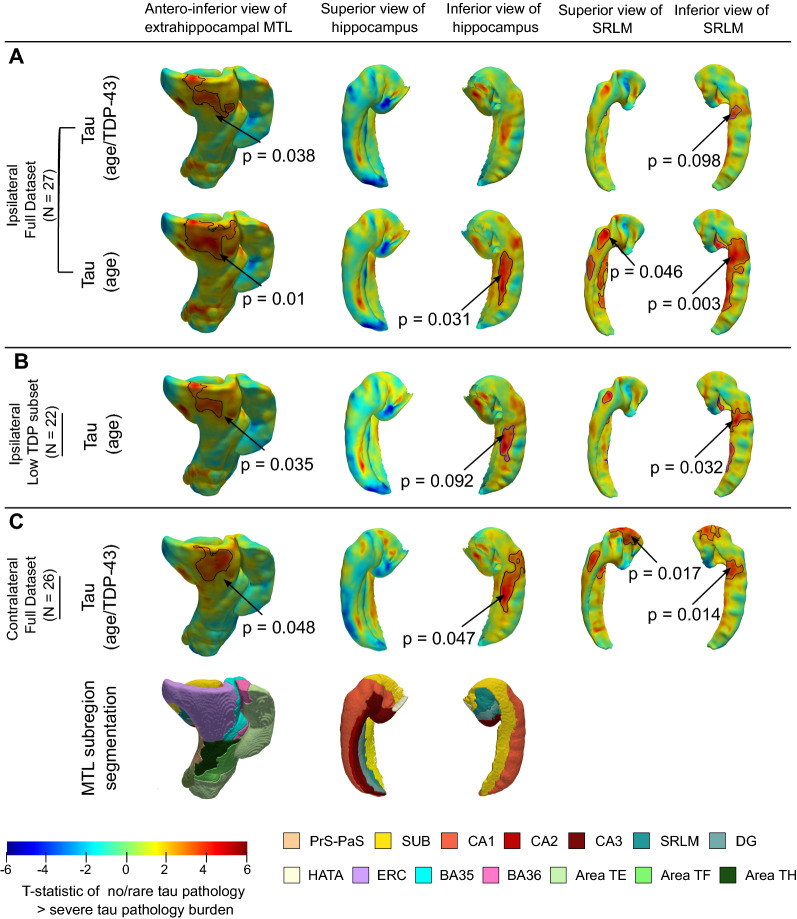
Statistical map of the correlation between cortical thickness and the severity of tau pathology. These analyses were performed in the subset of specimens age 59 years and older. The covariates used in each analysis are provided in parentheses. The clusters outlined in black indicate regions where a significant correlation was observed after correction for multiple hypothesis testing (corrected *p* < 0.05). The clusters outlined in blue indicate regions where a trend level correlation was observed (corrected *p* < 0.10). (*SUB* subiculum, *SRLM* stratum radiatum lacunosum moleculare, *CA* cornu ammonis, *DG* dentate gyrus, *HATA* hippocampal amygdala transition area, *ERC* entorhinal cortex, *BA* Brodmann Area)

Figure 4 shows the results of the pointwise thickness analysis performed on the MTL and SRLM examining the regional effects of tau pathology on cortical thickness using pathology ratings derived from the MTL ipsilateral to the thickness measures. Cases CNDR12 (44 y.o.) and HNL01 (45 y.o.) were excluded from the thickness analyses since these younger cases are outliers in terms of age and including them skews age effects, thereby dampening the associations between pathology and thickness. In this work we only consider the effects of tau and TDP-43 pathology on MTL structure since recent work studying the contribution of mixed pathology to MTL atrophy in AD showed no clear relationship between neurodegeneration and either Aβ or $\alpha$-synuclein [6]. The analysis considering both tau and TDP-43 pathology in the model reveals a significant association between tau rating and atrophy in the ERC region (corrected *p* = 0.038) with a smaller effect at a trend level in the SRLM (corrected *p* = 0.098). When using only age as a covariate (i.e., excluding TDP-43 from the model), stronger associations between tau pathology and thickness are observed in the ERC region, extending towards the transentorhinal cortex (corrected *p* = 0.01) and SRLM (inferior cluster: corrected *p* = 0.003, superior cluster: *p* = 0.046). Additionally, a significant cluster is observed in the SUB/CA1 region (corrected *p* = 0.031). To evaluate the effects of tau in the absence of severe TDP-43 pathology, regional thickness analysis was repeated in the subset of twenty-two specimens with low levels of TDP-43 pathology (average MTL TDP-43 rating < 1), using only age as a covariate. Once again, significant associations are found between tau and thickness in the ERC region (corrected *p* = 0.035) and SRLM (corrected *p* = 0.032), with a weakened association in the SUB/CA1 region (corrected *p* = 0.092). To further understand the weakened tau effects in the presence of TDP-43 pathology, we performed a supplementary analysis looking at effects of TDP-43 pathology on regional thickness with age and tau as covariates. Our analysis reveals significant associations between TDP-43 rating and atrophy in the posterior parahippocampal cortex (corrected *p* = 0.027), anterior hippocampus (corrected *p* = 0.01) and SRLM (inferior cluster: corrected *p* = 0.003, superior cluster: *p* = 0.011) (Additional file 1: Fig. S5).

### Evaluating neuropathological asymmetry

**Fig. 5:**
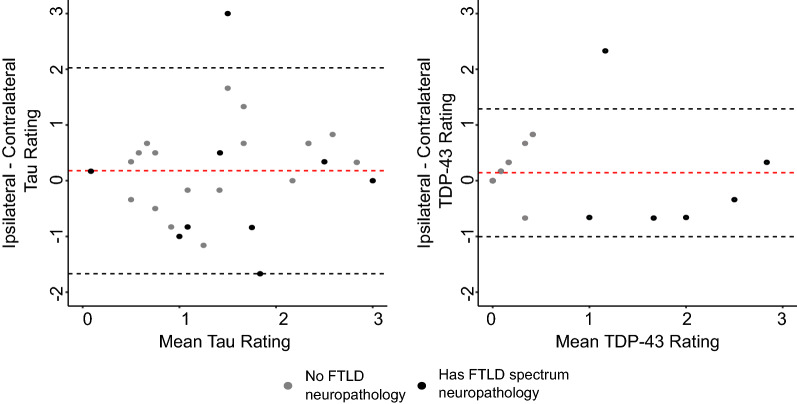
Bland–Altman plots showing the level of agreement between average ipsilateral and contralateral ratings of tau and TDP-43 pathology. The dashed red lines indicate the mean of the differences and the dashed gray lines indicate two standard deviations above and below that. A different color is used to indicate cases with frontotemporal lobar degeneration (FTLD) neuropathology. The raw ipsilateral and contralateral ratings for tau and TDP-43 pathology in each specimen are plotted in Additional file 1: Fig. S8. (*SUB* subiculum, *SRLM* stratum radiatum lacunosum moleculare, *CA* cornu ammonis, *DG* dentate gyrus, *HATA* hippocampal amygdala transition area, *ERC* entorhinal cortex, *BA* Brodmann Area)

For a subset of twenty-eight specimens, neuropathological examinations were performed in the hemisphere both ipsilateral and contralateral to the MTL that was scanned. Unlike the contralateral neuropathological examinations, which were performed for use in a clinical setting, the ipsilateral ratings are based on thicker histology sections intended for 3D serial reconstruction and are more experimental. Figure 5 shows Bland–Altman plots comparing average ipsilateral and contralateral ratings of tau and TDP-43 pathology. Cases with an FTLD neuropathological diagnosis are indicated using a different color since these cases are known to demonstrate prominent asymmetry [34, 35]. For most cases, the ipsilateral and contralateral ratings are consistent with each other. In five cases, there is some asymmetry in tau ratings (difference in average rating > 1). As expected, the largest discrepancies in ratings are observed in cases with FTLD neuropathology. CNDR15 demonstrates severe asymmetric tau burden (ipsilateral—3, contralateral—0) and has a neuropathological diagnosis of FTLD-TDP. Similarly, CNDR06 demonstrates significant asymmetry in TDP-43 pathology, with a difference of 2.33 in average rating between both sides and has an FTLD-tau neuropathological diagnosis.

In a secondary analysis, we were interested in using this data to evaluate the degree to which differences between ipsilateral and contralateral protocols, as well as hemispheric asymmetry and potential sampling effects, impact the results of our cortical thickness analyses. Figure 4 includes the result of the regional thickness analysis using contralateral pathology data, correlating cortical thickness and tau pathology with age and TDP-43 pathology as covariates. We observe patterns of correlation between thickness and tau pathology consistent with the ipsilateral analysis (cluster in ERC, corrected *p* = 0.048), and associations in the SRLM (corrected *p* = 0.014 and 0.017) and SUB/CA1 region (corrected *p* = 0.047) that are stronger in absolute terms than in the ipsilateral analysis. Similar patterns of correlation are also observed between thickness and contralateral ratings of TDP-43 pathology when compared to the results of the ipsilateral TDP-43 analysis (Additional file 1: Fig. S5).

## Discussion

Using a highly customized registration framework, we generated a first-of-its-kind 3D probabilistic atlas of the human MTL that combines ultra-high-resolution ex vivo MRI scans of 29 specimens, with histology-based annotations in 11 specimens from a cohort of brain donors that includes patients with AD and related dementias, as well as neurologically unimpaired individuals. In prior work, we generated an ex vivo probabilistic atlas of the human hippocampus [28]. Here, we show that the same atlas construction framework can be optimized to co-register the extrahippocampal regions which present a separate set of challenges for groupwise alignment due to significant variability in patterns of cortical folding [32, 36]. In contrast to [28], where clinical diagnosis of AD was used to characterize the effects of AD on the hippocampus, we use neuropathological ratings to more specifically characterize the effects of tau and TDP-43 pathology on MTL morphometry. Leveraging this atlas and histology-based pathology measures, we are now able to provide a direct link between changes in MTL structure and the underlying neurodegenerative pathologies to identify specific regions in the MTL where patterns of structural change are associated with the severity of tau pathology during the early stages of the disease.

Early works by Braak and Braak describe a characteristic pattern of spread of NFTs within the brain which encompasses six stages [1]. The first three Braak stages are defined by the stereotypical pattern of involvement of specific MTL regions, suggesting differential vulnerability of these regions to early tau pathology and neuronal loss. When looking at the relationship between cortical thinning and ratings of tau severity, after accounting for age, our analysis reveals significant correlations in the ERC and SUB/CA1 region of the MTL, consistent with the earliest regions affected by NFTs based on early Braak staging. We also observe significant associations in the SRLM, in agreement with histopathology studies showing early involvement of tau pathology in the SRLM of CA1, as well as prior in vivo MRI studies which have demonstrated SRLM atrophy in patients with AD [9, 28, 37, 38]. Similar associations, albeit weakened, are observed when we perform thickness analysis in the subset of specimens with low levels of TDP-43 pathology (average MTL TDP-43 rating < 1) further confirming the specificity of atrophy in these regions to tau pathology.

Interestingly, this set of locations is also in agreement with the results of an in vivo study that analyzed the association between ^18^F-flortaucipir uptake in the MTL and atrophy [21]. Although tau PET is limited by its spatial resolution and confounds such as off-target binding [39], the matching patterns of atrophy provide support for the specificity of ^18^F-flortaucipir uptake to underlying tau pathology. However, in our thickness analysis, we find that after accounting for the presence of co-existing TDP-43 pathology in the full dataset, the associations between tau and thickness are weakened in the ERC and SRLM, while the SUB/CA1 associations no longer reach significance. In the supplementary analysis looking at the effects of TDP-43 pathology on MTL atrophy, we observe significant associations between cortical thinning and TDP-43 pathology in the anterior hippocampus, SRLM and posterior PHC region. Our finding of TDP-43 effects in the anterior hippocampus is consistent with previous studies [6, 40]. The strong TDP-43 effects suggest that by covarying for TDP-43 pathology in our analysis, we may be obscuring some of the associations due to tau pathology, and vice versa. We note that for one of the cases included in the study (CNDR06), which demonstrates severe tau and TDP-43 pathology, the ex vivo MTL scan displayed significantly less atrophy when compared to the donor’s antemortem in vivo MRI scan. We hypothesize that this is likely due to brain swelling. Additional file 1: Fig. S6 shows the results of the primary analysis with this specimen excluded, with similar patterns of tau/thickness correlations (with age and TDP-43 pathology as covariates) but weakened significance, likely explained by the fact that despite the swelling, this case had lower than average thickness, and removing the case from the analysis reduced the degrees of freedom in the analysis. While this issue was not observed in the remainder of the dataset (16 of the 29 ex vivo scans had antemortem MRI available), further research needs to be done to understand how to account for this potential source of variability.

Our finding of tau effects in the anterior ERC and SUB/CA1 region appears inconsistent with an antemortem study by de Flores et al. [6] that correlated antemortem MTL subregional volumes with semi-quantitative tau pathology ratings in the MTL as well as global Braak staging scores and found a significant association between tau and the posterior hippocampus but no other MTL subregions. It is important to note that there are significant differences in the study population; the de Flores et al. study consisted of a larger dataset with a greater number of TDP-43 positive cases which may have concealed tau effects in anterior MTL regions. Indeed, the heat maps in Fig. 4 in the de Flores et al. study show hints of correlation with tau in the anterior MTL, but these do not reach significance at a whole ROI level. Surprisingly we do not detect posterior hippocampal tau effects in the current study. This may be due to the small sample size, the fact that de Flores et al. only included cases with at least low levels of AD neuropathologic change in their study, and the limited number of cases with late-stage AD in the present study. The stronger posterior effect in de Flores et al. could be explained in part by the larger number of cases in their study with high AD neuropathologic change, since advanced AD is associated with more diffuse tau burden, spreading away from anterior MTL structures [41]. Once our dataset grows to include more pathology specimens, we will be able to better examine this by studying the evolution of NFT-specific “hotspots” as AD pathology progresses.

On the contrary, our results complement the recent postmortem study by Wisse et al. [25] that measured MTL thickness at seven hand-picked locations and reported associations with clinical neuropathology measures from the contralateral hemisphere. Wisse et al. found significant associations between tau pathology and the thickness of BA35, SRLM and ERC (at a trend level), and widespread associations between TDP-43 pathology and almost all MTL subregions. The current analysis includes twenty-six of the fifty-eight specimens in [25] plus three additional specimens (the inclusion criteria for our morphological study are more stringent than [25], requiring good quality MRI signal over most of the MTL extent); and it uses ipsilateral as well as contralateral pathology measures. The computational atlas of the MTL allows us to study patterns of thinning associated with neurodegenerative pathologies at a much more fine-grained level than Wisse et al. [25]. Indeed, our results show refined patterns of atrophy specific to tau pathology in the ERC and SRLM, consistent with [25]. Unlike [25], we do not observe tau effects in BA35. We note that given the early involvement of NFTs in BA35, one would expect to see stronger correlation patterns along the medial bank of the collateral sulcus. While our groupwise registration approach enables reliable quantification of thickness in most specimens, accurate characterization of the collateral sulcus during atlas construction is challenging due to large anatomical variability and complex sulcal geometry [33]. Therefore, unresolved misalignments between odd sulcal patterns in some specimens likely weaken the power of measurements fully within BA35 and the ability to detect associations in this region.

**Fig. 6:**
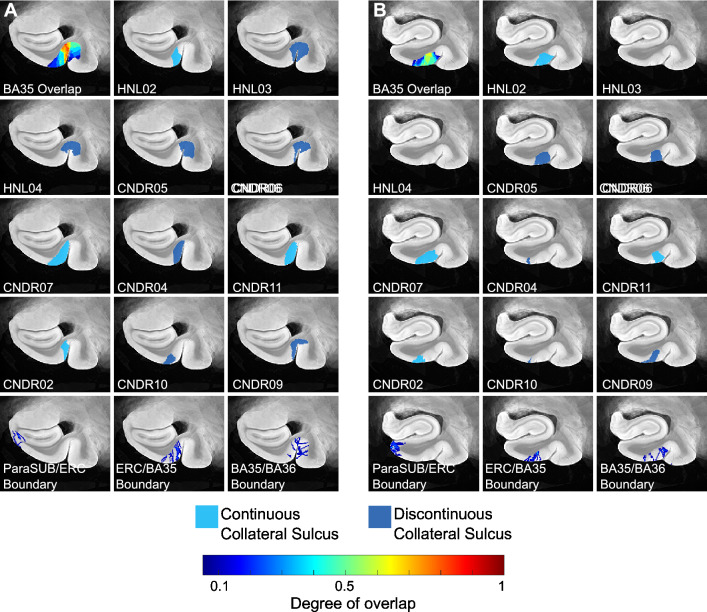
Location of medial temporal lobe (MTL) subregion, Brodmann Area 35 (BA35), defined histologically in eleven specimens and mapped into the space of the MRI atlas. Different color labels are used to indicate the type of sulcal pattern each speciemen has: type 1, deep continuous sulcus; type 2, discontinuous sulcus with shallower anterior branch. Panels **A** and **B** each show a cross-sectional view through the MTL at an anterior and more posterior level respectively. Each panel includes a heat map (top-left corner) showing the degree of overlap across the different specimens. The bottom row demonstrates the variability in the location of specific anatomical bondaries between different specimens. Note that boundaries in which a background label is adjacent to a non-background label are not considered, i.e. in some cases, the ERC label is adjacent to the background instead of the para-subiculum. (*ERC* entorhinal cortex, *ParaSUB* para-subiculum, *BA* Brodmann Area)

Furthermore, the weakened associations in BA35 could be partially attributed to variability in the location of BA35 within the MTL cortex between specimens. Prior studies have reported that the anatomical extents of BA35 and BA36 are highly dependent on the depth and branching pattern of the anterior collateral sulcus (CS) [32, 33]. In specimens with a deep continuous sulcus, BA35 occupies part of the medial bank of the CS, whereas in specimens with a discontinuous sulcus, where the depth of the anterior branch is generally shallow, BA35 occupies a more superior portion of the medial bank, and even goes over the fundus to cover the lateral bank of the sulcus up to the midpoint of the fusiform gyrus in some cases. A similar pattern is observed in our dataset, as shown in Fig. 5 which illustrates the location of BA35 in the space of the ex vivo atlas for the eleven specimens with histologically derived MTL subregion labels. Although the registration pipeline deforms each specimen’s anatomy to create an average sulcus morphology, we notice that in the atlas space, for cases with a discontinuous sulcus, BA35 tends to start further along the medial bank of the CS and extends over the fundus. On the other hand, for cases with a continuous CS, BA35 only occupies the medial bank of the CS. Figure 6 also plots heatmaps indicating the overlap between BA35 across all specimens as well as the anatomical boundaries between the SUB/ERC, ERC/BA35 and BA35/BA36. We see that the boundaries between ERC/BA35 and BA35/BA36 are highly dispersed and span the entire length of the CS. In contrast, the SUB/ERC boundary is not as variable. In general, the boundary dispersion is greater in the anterior portion of the MTL, likely due to greater variability in sulcal depth in this region. While our registration approach significantly improved atlas quality compared to a more conventional method, a more advanced computational method which explicitly enforces cortical geometry needs to be developed to fully address this problem.

In a secondary analysis, we investigated the degree to which hemispheric asymmetry impacts the findings of our regional thickness analyses using data from the twenty-eight donors for whom we had neuropathological ratings sampled from both hemispheres. Previous studies have shown some asymmetry in ratings of tau pathology, and TDP-43 in 15–43% of cases with AD [42, 43]. In our dataset, we observe discrepancies in the ratings of tau pathology between both sides in 18% of cases, with the largest discrepancies in the average ratings of tau and TDP-43 pathology in cases with a neuropathological diagnosis of FTLD-TDP and AGD respectively. Pathological asymmetry is expected in these types of cases with FTLD neuropathology. It is important to note that differences in sampling location, protocols and staining used to obtain the ipsilateral and contralateral ratings may be contributing to some variability between the two sides. Despite these differences, the regional thickness analysis performed using the contralateral ratings reveals significant associations between MTL thickness and tau/TDP-43 pathology in regions consistent with the results of the ipsilateral analysis. Although one might expect the correlations to be weaker when using contralateral ratings, the stronger correlations may be explained by the observation that several of the cases in our dataset that have a contralateral TDP-43 rating of zero, tend to have an ipsilateral TDP-43 rating in the range of zero to one which is considerable on a scale from 0 to 3. This variability in ratings between the two sides is likely due to the fact that the contralateral ratings are derived using a more clinical protocol unlike the ipsilateral ratings, which are more experimental.

The limitations of the present study are the clinical heterogeneity of our dataset and the reliance on semi-quantitative ratings of tau and TDP-43 pathology. The semi-quantitative measures are sensitive to inter-rater and intra-rater errors and likely contribute to some uncertainty in our findings [44]. Additionally, current ratings of tau and TDP-43 pathology do not distinguish between the various subtypes of pathology. Tau pathology takes multiple forms, including tangles, pre-tangles, FTLD-tauopathy, or aging-related tau astrogliopathy (ARTAG) [45]. Likewise, there are distinct subtypes of TDP-43 pathology (neuronal cytoplasmic inclusions, neuronal intranuclear inclusions, dystrophic neurites, white matter threads), which may be linked to different clinical manifestations of neurodegenerative disease [46]. Although the current measures do not assess NFTs specifically, we believe that the patterns of atrophy identified in our analyses likely reflect NFT-related changes since they are consistent with the regions affected early in Braak staging. Moreover, as per the B scores provided in Additional file 1: Table S2, Braak I, or higher stage tau pathology was observed in at least 69% of our specimens, indicating the presence of NFTs specifically. Example patches of ipsilateral tau IHC sections sampled at the locations corresponding to the AD-specific “hotspots” found in Fig. 4B are shown in Additional file 1: Fig. S10. By visual assessment, we can observe some tangles among the specimens without primary FTLD-Tau or AGD. However, more evidently, we observe a trend of increased neuropil threads, which are a part of Braak staging [47], as cortical thickness in the region of the hotspot decreases. Furthermore, we note that the cases with primary FTLD-Tau/AGD are evenly distributed between cases with high and low thickness, particularly in the SUB and SRLM hotspots, suggesting that FTLD-associated tau is unlikely to be driving the tau-structure associations in these regions. To further examine the contribution of NFTs to neurodegeneration in the hotspots, we performed a preliminary analysis in a subset of fifteen specimens for which 3D quantitative maps of NFT burden derived from dense serial histology are available [29]. These NFT density maps are generated using a weakly supervised deep learning algorithm, trained to specifically detect tangles and pre-tangles on AT8-stained sections and have been shown to correlate strongly with manual NFT counts [29]. Three cases with a primary FTLD-Tau or AGD diagnosis (CNDR01, CNDR06 and CNDR07) were excluded from the analysis since they likely contain 4R-tau inclusions which can be difficult to distinguish from AD-related NFTs. In the remaining twelve cases, we examined the correlation between the average NFT burden computed within each hotspot and the median thickness of each hotspot, and observe very strong correlations, although the hotspot in the ERC does not reach significance (Additional file 1: Fig. S12). These results are weakened when we include age in the model. This is likely due to the small sample size and the fact that age and tau are significantly correlated (average R = 0.61 across the three hotspots). Despite the small dataset, these results are encouraging and suggest that NFTs are indeed playing a role in driving neurodegeneration in the ERC, SUB and SRLM hotspots. As we expand our dataset, collect more quantitative histology data and develop automated methods to extract quantitative measures of the different types of tau and TDP-43 pathology, in future work we will be able to better overcome the above-mentioned limitations and validate the relationship between NFTs and thickness.

Overall, our findings provide a more refined understanding of how tau pathology is associated with cortical thinning within the MTL and motivate further characterization of the MTL in AD using detailed ex vivo MRI analysis. The clusters identified from the tau thickness analyses indicate granular MTL regions where in vivo measures of neurodegeneration are expected to be strongly associated with tau pathology. In an exploratory analysis, we attempted to use the ex vivo derived, AD-specific hotspots (Fig. 4B) as biomarkers in a longitudinal analysis of ADNI data (not shown). While the hotspots did not show statistical effects in a group comparison of Aβ negative, tau negative (A−T−) and Aβ positive, tau positive (A+T+) patient groups with mild cognitive impairment, BA35 achieved the strongest group discrimination. The lack of statistically significant group differences with the hotspots may be due to the small ex vivo sample size, clinical heterogeneity in our dataset and difficulties in accurately aligning BA35 in the atlas.

Constructing a probabilistic atlas of the MTL has far-reaching applications in AD research beyond the work shown here. Perhaps future analyses leveraging this technology in a patient cohort more consistent with the one we would expect to encounter in an AD clinical trial would result in hotspots that are more sensitive to longitudinal change in the presence of NFT pathology, potentially enabling the development of neurodegeneration biomarkers which are more effective during early AD clinical trials. Furthermore, in future work, 3D quantitative maps of NFT density derived from serial histology imaging will be mapped into ex vivo atlas space to generate a comprehensive probabilistic description of the progression of NFT pathology at each Braak stage [29, 48, 49]. This will allow us to describe NFT topography during the different stages of AD in more detail than current descriptions, which are in 2D and based on selective sampling of the MTL [1, 2]. Additionally, in a future version of the atlas, cytoarchitecture-guided anatomical labels of MTL subregions will be included from a larger number of specimens. Such an atlas would reflect anatomical ground truth and can be used to inform in vivo MRI protocols for segmentation of MTL subregions, thereby improving the accuracy of MRI biomarkers derived from these subregions.

## Supplementary Information

**Additional file 1.** Supplementary methods and results.

## Data Availability

Anonymized MRI data and intermediate results have been uploaded to https://openneuro.org/ (dataset https://doi.org/10.18112/openneuro.ds003052.v1.1.0). Source code used to construct the atlas and for statistical analysis can be accessed via GitHub (sadhana-r/Penn_exvivoMTLAtlas.git).
