# Supplementary material for: Ex vivo MRI atlas of the human medial temporal lobe: characterizing neurodegeneration due to tau pathology
Source: Acta Neuropathol Commun. 2021 Oct 24;9:173. doi: 10.1186/s40478-021-01275-7 (PMC8543911; doi:10.1186/s40478-021-01275-7)
Supplement: Supplementary file 1 — Additional file 1. Supplementary methods and results. [file 40478_2021_1275_MOESM1_ESM.docx]

Supplementary Material for

**Ex vivo MRI atlas of the human medial temporal lobe: characterizing neurodegeneration due to tau pathology**

Sadhana Ravikumar*^a,b^, Laura E.M. Wisse_­_^b,c,d^, Sydney Lim^b^, Ranjit Ittyerah^b^, Long Xie^b^, Madigan L. Bedard^b^, Sandhitsu R. Das^c^, Edward B. Lee^e^, M, Dylan Tisdall^b^, Karthik Prabhakaran^b^, Jacqueline Lane^c^, John A. Detre^c^, Gabor Mizsei^b^, John Q. Trojanowski^e^, John L. Robinson^e^, Theresa Schuck^d^, Murray Grossman^b^, Emilio Artacho-Pérula^e^, Maria Mercedes Iñiguez de Onzoño Martin^f^, María del Mar Arroyo Jiménez^f^, Monica Muñoz^f^, Francisco Javier Molina Romero^f^, Maria del Pilar Marcos Rabal^f^, Sandra Cebada Sánchez^f^, José Carlos Delgado González^f^, Carlos de la Rosa Prieto^f^, Marta Córcoles Parada^f^, David J. Irwin^c^, David A. Wolk^c^, Ricardo Insausti^f^, Paul A. Yushkevich^a,b^

^a^Department of Bioengineering, University of Pennsylvania, Philadelphia, PA 19104, USA

^b^Department of Radiology, University of Pennsylvania, Philadelphia, PA 19104, USA

^c^Department of Neurology, University of Pennsylvania, Philadelphia, PA 19104, USA

^d^Department of Diagnostic Radiology, Lund University, 221 85 Lund, Sweden

^e^Department of Pathology, University of Pennsylvania, Philadelphia, PA 19104, USA

^f^Human Neuroanatomy Laboratory, CSIC Neuromax Associated Unit, University of Castilla La Mancha, 02008 Albacete, Spain

**1. Supplemental Methods**

- 1. **Specimens and postmortem MRI**

Specimens were scanned in a cylindrical container containing an MRI-neutral industrial lubricant. The specimens were imaged with a custom-made 70 mm coil, using an acrylic holder with 49.8 mm inner diameter and a long (80 mm) z-field^[[1]](#footnote-1)^. Prior to scanning, special precautions were taken to eliminate air bubbles trapped in both the brain and container. To reduce the chance of having air bubbles trapped along the cortical surface, the specimens were gently agitated by hand while submerged in the MRI-neutral lubricant. MRI scans were obtained using a multi-slice spin echo sequence. Sequence parameters varied slightly between specimens, with typical values being a repetition time of 9330 ms, and an echo time of 23 ms. Following image acquisition, the images were corrected for bias field non-uniformity using the N4ITK algorithm [11] and normalized to a common intensity range by clipping the intensities below the 0.1 and above the 99.9 percentile, and scaling the intensity range to [0,1000]. Due to an error in the scanner gradient calibration, as part of the post-processing, all of the scans had to be linearly scaled (6% in x, 3% in y, 11% in z) to correct for differences between the scanner coordinate frame and physical coordinate frame. These linear scaling factors were derived using a 3D printed phantom [1]. To study the effects of the applied scaling factors on the ex vivo analysis correlating the severity of neurodegenerative pathologies with regional thickness**,** the analysis was repeated using native space measurements. *Supplementary Fig. 7* plots the associations between thickness, computed in native space, and ratings of the severity of tau pathology. In general, similar correlation patterns between MTL atrophy and tau pathology are observed in both the native and distortion-corrected image space, indicating that the findings of our ex vivo thickness analysis are likely not affected by the applied linear scaling factors.

- 1. **Ex vivo MTL and SRLM segmentations**

Segmentations of the medial temporal lobe (MTL) and stratum radiatum lacunosum moleculare (SRLM) were used to guide groupwise registration. To generate the MTL segmentations, a semi-automated interpolation method was developed and implemented in ITK-SNAP software [15]. As a first step, the boundary of the MTL was manually traced approximately every 15 slices in each of the twenty-four specimens. In some of the specimens, parts of the MTL were not visible due to variability in the amount of tissue cut and imaged. Therefore, a segmentation protocol was developed to ensure consistency across specimens. The segmented region includes the hippocampus, entorhinal cortex (ERC), portion of the perirhinal cortex (PRC) up to the medial bank of the occipitotemporal sulcus (which served as the anterior lateral border), and parahippocampal cortex (PHC) (the fundus of the collateral sulcus served as the posterior lateral border). In two specimens, the anterior lateral border for the PRC could not be extended past the lateral edge of the collateral sulcus. Given the subset of labelled slices, the interpolation method uses binary weighted averaging and a random forest classifier to compute the intermediate segmentations [9]. This algorithm was applied iteratively, allowing the interpolated result to be reviewed and manually edited at each step to refine the segmentation. The segmentations were performed by author SR and checked by author LW. Multi-label segmentations for sulci delineation and image artifacts were incorporated after generating the complete 3D segmentations.

In four of the specimens, the SRLM was labelled using the approach described in Adler et al. [1]. In the remaining twenty-five cases, the SRLM segmentations were initialized using a supervised deep learning approach and completed using manual editing in ITK-SNAP. First, we developed a neural network to perform pixel-wise segmentation using data from the 31 specimens used in [1] for which hippocampus/SRLM segmentations have been previously completed. The network was trained on over 9000 3D image patches, extracted from 26 of these datasets, and the remaining 5 datasets were used to validate the network’s performance. For the network architecture, we adapted the widely used 3D U-Net approach and incorporated deep supervision [5, 10, 16]. Given an input image patch, the network yields a segmentation classifying each voxel as either background, hippocampus or SRLM. To further improve the performance of this method, we extracted the largest connected SRLM component from the predicted segmentation. The output of this approach was then manually edited to obtain the final SRLM segmentation. Example MTL and SRLM segmentations obtained from these procedures are shown in *Supplementary Fig. 1.*

- 1. **Atlas construction**

The atlas construction pipeline consists of three stages, as described in Adler et al. [1]. The first stage uses shape matching via minimum spanning trees (MST) to identify a registration sequence that minimizes the amount of shape deformation that is required to achieve a common image space across all the specimens. The MST stage yields a set of affine and deformable transformations between each specimen and a common “root” specimen, which closely matches up the boundaries of the medial (red label shown in *Supplementary Fig. 1)* and lateral (blue label shown in *Supplementary Fig. 1)* segments of the MTL and the SRLM. The average shape of the MTL/SRLM following this stage is biased by the anatomy of the root specimen. Therefore, the next stage of the pipeline performs shape correction using Geodesic Shooting (GS). The shape averaging algorithm iteratively corrects the MTL shape by alternating between registering each specimen to the shape average using a combination of rigid alignment and geodesic shooting [2, 12], and updating the shape average by applying geodesic shooting in the direction of the average initial momenta. This process was repeated for five iterations until the shape average stabilized. For the final stage, a population template is generated by performing intensity-based deformable registration (INT) using the groupwise unbiased framework proposed in [6] and the normalized cross-correlation (NCC) metric. Unlike the previous stages which rely predominantly on the MTL and SRLM segmentations for shape information, this stage incorporates intensity information from the MR images to resolve residual misalignments between specimens. During this stage, we introduced the capability to handle image artifacts by using the “artifact label” (green label shown in *Supplementary Fig. 1*) to mask out affected regions from intensity-based registration computations. For more details on the atlas construction pipeline, see Supplemental Information S1.3 in Adler et al. [1]. An updated table of registration parameters used at each stage of the pipeline is provided in *Supplementary Table 1*. The output of this algorithm is a template MR image, the corresponding template segmentation and a set of non-linear diffeomorphic transformations between the template and each individual specimen’s scan. To generate a binary MTL segmentation, semi-automated active contour segmentation using ITK-SNAP [15] was applied to the average of the warped segmentations of all specimens using a threshold of 0.65. This resulted in a smoother and more geometrically accurate final segmentation for morphometry analysis compared to simply thresholding the average warped segmentation generated by the pipeline. A binary SRLM segmentation was generated by thresholding the average of the warped SRLM segmentations of all specimens at 0.45.

- 1. **Computation of Regional MTL Thickness**

To evaluate patterns of cortical thinning, a surface mesh was extracted from the template segmentation generated by the atlas pipeline using the marching cubes algorithm [7]. The surface was smoothed using the Taubin algorithm implemented in MeshLab to remove sharp edges [3]. Using transformations from the atlas pipeline, this smoothed mesh was then warped into the space of each specimen. For each specimen, regional thickness was measured by first extracting the pruned Voronoi skeleton [8] of the warped template mesh and computing twice the distance between each vertex and the closest point on the skeleton. Thickness measurements were then brought back into the atlas space, resulting in a set of twenty-nine observations at each vertex. Due to the image artifacts described previously, at certain vertices in some specimens, thickness measurements had to be excluded from the mesh. This resulted in a variable number of observations at each vertex and was accounted for in the statistical analysis.

**1.5 Mesh-based statistical analysis**

Statistical analyses correlating regional thickness with age, tau and TDP-43 pathologies were performed using the open-source tool ‘meshglm’ (<https://github.com/pyushkevich/cmrep/>). Using this method, we fit a general linear model (GLM) at each vertex on both the MTL and SRLM surfaces with age or the average rating of the pathology of interest as the independent variable, thickness as the dependent variable, and the rating of the other proteinopathy and/or age as covariates. Before statistical analysis, spatial smoothing (diffusion) is applied to the thickness data using a simple implementation of the heat equation (diffusion parameter, T = 4). The diffusion method was modified to avoid propagation of missing thickness values. Additionally, the statistical computation was adapted to handle missing data by accounting for the variable number of observations and therefore degrees of freedom at each vertex. To account for multiple comparisons, the analysis uses cluster-level family-wise error rate correction [19]. Potential clusters were defined based on an empirical threshold (uncorrected p <= 0.01) and permutation testing with the Freedman & Lane method (1000 iterations) was used to assign each potential cluster a corrected p-value [13].

1. **Supplemental Results**
   1. **MRI Atlas Quality**

*Supplementary Fig. 2* shows the atlas obtained following each stage of the atlas construction algorithm, as described in Section S1.3. Atlases labeled “MST”, MST+GS”, and “MST+GS+INT” correspond to the three stages of the atlas pipeline, specifically minimum spanning tree, geodesic shooting and intensity-based groupwise registration. Comparing the “MST + GS” and “MST” stages, we can see a visible difference in the shape of the atlas due to the shape averaging effect of the geodesic shooting algorithm. We also observe that the final stage of incorporating intensity information (“MST+GS+INT”) results in noticeably sharper intensity features. Atlas “INT only” shown in the bottom row of *Supplementary Fig. 2* serves to demonstrate that the more conventional approach of groupwise intensity-based deformable registration, typically used in in vivo morphometry studies, does not work as well as the developed multi-step pipeline. The dashed red circles in the figure highlight regions where the “MST+GS+INT” atlas achieves a markedly better result than the “INT only” atlas. In general, the developed pipeline is able to better align the head of the hippocampus as well as the highly variable collateral sulcus region of the MTL, resulting in a more defined structure.

*Supplementary Fig. 3* plots quantitative metrics of atlas quality following each of the three stages of atlas construction, and the “INT only” approach. After each stage, we computed the average Dice similarity coefficient (DSC) for the entire MTL segmentation across all pairs of specimens registered to a given atlas [4], and the average NCC metric between each pair of specimens registered to the atlas, integrated over the volume of the MTL segmentation. The first two stages of atlas construction directly use segmentations of the MTL to guide registration. Therefore, it is to be expected that “MST” and “MST+GS” would result in higher DSC metrics. Similarly, the “MS+GS+INT” step achieves a higher NCC metric since it incorporates intensity-based registration which optimizes the NCC metric. The key observation in this figure is that the final atlas output (“MST+GS+INT”) achieves significantly better MTL overlap (higher DSC value) and a greater NCC metric across all pairs of specimens compared to the conventional “INT only” approach. This shows that the initialization, provided by the “MST+GS” stages, helps intensity-based registration find a better solution compared to initialization based on groupwise affine registration only. *Supplementary Fig. 4* shows cross-sectional views of the voxel-wise average NCC metric between each pair of specimens registered to the atlas following the final stage of the pipeline, as well as the average of the segmentations of all specimens registered to the atlas. In general, there is strong agreement along the edges of the MTL. From this figure we can see that the quality of registration is poorest in the regions surrounding the collateral sulcus. While the MTL and SRLM overlap is close to one along the length of most of the MTL, in a few specimens, odd sulcal patterns make accurate registration challenging, particularly towards the anterior MTL.

**Supplemental Figures**


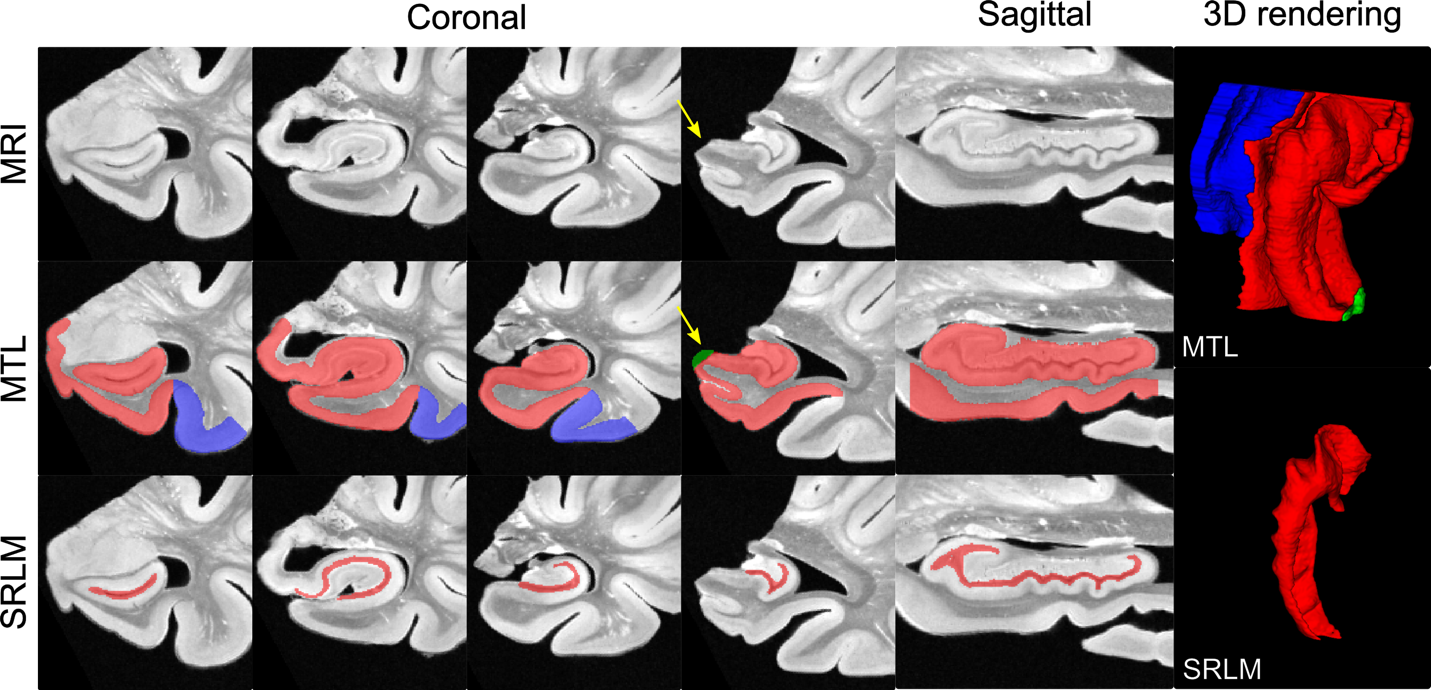


**Supplementary Figure 1:** **Example semi-automatic segmentation** **of the medial temporal lobe (MTL) and stratum radiatum lacunosum moleculare (SRLM) in an ex vivo MRI scan. These segmentations were used to guide groupwise registration for atlas construction**. In some specimens, the collateral sulcus is tightly folded, making it difficult to distinguish the medial and lateral banks of the sulcal fold. To address this, a separate label was used to label the lateral portion of the collateral sulcus (blue). The yellow arrow provides an example of a minor tear in the tissue. A green label was used to indicate minor regions of the image with MRI artifacts, cut or missing tissues, or other irregularities.

| **Stage of pipeline** | **Registration step** | **Parameter** | **Value** |
| --- | --- | --- | --- |
| **Shape Matching via Minimum Spanning Tree (MST)**  **(Refer to Adler et al. SI Section S1.3.2)** | $A_{i\to j}^{0}$ | $\mu$ | NMI |
|  |  | $w$ | {1,2,5} |
|  | $A_{i\to j}^{rough}$ | $\mu$ | SSD |
|  |  | $w$ | {1,2,5} |
|  | $\emptyset_{i\to j}^{rough}$ | $\mu$ | NCC [2x2x2] |
|  |  | $w$ | {1,2,5} |
|  |  | $\sigma_{s}$ | 2 mm |
|  |  | $\sigma$ | 0.1 mm |
|  |  | $N$ | {100,80,80,0} |
|  | $A_{i_{m}\to i_{m-1}}^{mst}$ | $\mu$ | SSD |
|  |  | $w$ | $\{1,2,5\}$ |
|  | $\emptyset_{i_{m\to i_{m-1}}}^{mst}$ | $\mu$ | SSD |
|  |  | $w$ | $\{1,2,5,\epsilon_{\Upsilon}=7.{10}^{-8}\}$ |
|  |  | $\sigma_{s}$ | 0.6 mm |
|  |  | $\sigma$ | 0.1 mm |
|  |  | $N$ | {100,80,50,30} |
| **Shape Averaging via Geodesic Shooting (GS)**  **(Refer to Adler et al. SI Section S1.3.3)** |  | Number of Iterations | 5 |
| **Groupwise Intensity Based Registration**  **(Refer to** **Adler et al. SI Section S1.3.4)** | $\emptyset_{j}^{int,m}$ | $\mu$ | NCC[5x5x5] |
|  |  | $\sigma_{s}$ | 0.6 mm |
|  |  | $\sigma$ | 0.2 mm |
|  |  | $N$ | {100x100x50} |
|  |  | Number of Iterations | 6 |

**Supplementary Table 1:** **Registration parameters used during the atlas construction pipeline.** The notation and variables used here correspond to the equations described in the Supplemental Information (SI) S1.3 of Adler et al. [1]. The weight factors, $w$ refer to the weights given to the medial and lateral segmentations of the medial temporal lobe (MTL), and the stratum radiatum lacunosum moleculare (SRLM) i.e. {medial MTL, lateral MTL, SRLM}

| **Anonymization Number** | **Side** | **Age** | **Sex** | **Average Pathology Rating** | | **Neuropathological Diagnosis (Primary)** | **Neuropathological Diagnosis (Secondary)** | **Neuropathological Diagnosis (Tertiary)** | **A score** | **B score** | **C score** |
| --- | --- | --- | --- | --- | --- | --- | --- | --- | --- | --- | --- |
|  |  |  |  | **Tau** | **TDP-43** |  |  |  |  |  |  |
| CNDR01 | R | 76 | F | 3 | 0.83 | CBD | Low ADNC | - | 1 | 0 | 1 |
| CNDR02 | L | 76 | M | 1 | 0.33 | CVD | Low ADNC | - | 1 | 1 | 0 |
| CNDR03 | L | 65 | M | 0.5 | 0.67 | FTLD-TDP | PART | - | 0 | 1 | 0 |
| CNDR04 | R | 82 | F | 1.67 | 1.33 | FTLD-TDP | PART | - | 0 | 2 | 0 |
| CNDR05 | R | 70 | M | 1.33 | 0.17 | LBD | PART | - | 0 | 2 | 0 |
| CNDR06 | L | 80 | M | 2.67 | 2.33 | Agyrophilic Grain Disease | PSP | - | 1 | - | 0 |
| CNDR07 | R | 77 | M | 2.67 | 3 | CBD | FTLD-TDP | Low ADNC | 1 | 0 | 1 |
| CNDR08 | L | 79 | F | 0.67 | 0.17 | PSP | - | - | 0 | 0 | 0 |
| CNDR09 | L | 73 | F | 1 | 0 | Multiple system atrophy | CVD | Low ADNC | 1 | 1 | 0 |
| CNDR10 | L | 83 | M | 3 | 0.33 | LBD | Intermediate ADNC | - | 3 | 2 | 3 |
| CNDR11 | R | 59 | M | 1 | 1.67 | FTLD-TDP | Low ADNC | - | 1 | 2 | 0 |
| CNDR12 | L | 44 | M | 0.17 | 0 | Amyotrophic lateral sclerosis | - | - | 0 | 0 | 0 |
| CNDR13 | R | 73 | M | 3 | 0 | Pick’s disease | - | - | 0 | - | 0 |
| CNDR14 | R | 83 | F | 1.33 | 0.17 | PSP | Low ADNC | - | 1 | 0 | 0 |
| CNDR15 | L | 70 | M | 3 | 2.33 | FTLD-TDP | Low ADNC | - | 1 | 0 | 0 |
| CNDR16 | R | 71 | M | 3 | 0 | LBD | Intermediate ADNC | - | 3 | 2 | 1 |
| HNL01 | L | 45 | M | 0.5 | 0 | PART | - | - | 0 | 1 | 0 |
| HNL02 | L | 78 | F | 1.5 | 0 | Low ADNC | CVD | - | 1 | 1 | 1 |
| HNL03 | R | 93 | M | 2.17 | 0.33 | Intermediate ADNC | CAA | - | 3 | 2 | 3 |
| HNL04 | R | 71 | M | 0.67 | 0 | Low ADNC | - | - | 1 | 1 | 0 |
| HNL05 | R | 74 | M | 2 | 0.33 | PART | CAA | - | 0 | 2 | 0 |
| HNL06 | R | 61 | M | 0.5 | 0 | PART | CBD | - | 0 | 1 | 0 |
| HNL07 | R | 90 | M | 2.33 | 0.17 | Low ADNC | - | - | 1 | 1 | 0 |
| HNL08 | L | 66 | F | 0.5 | 0 | Part | Incidental LBD | - | 0 | 1 | 0 |
| HNL09 | R | 93 | F | 2.67 | 0.67 | Low ADNC | - | - | 1 | 1 | 1 |
| HNL10 | L | 90 | M | 2.33 | 0.83 | Low ADNC | Incidental LBD | - | 2 | 1 | 2 |
| HNL11 | R | 74 | M | 0.83 | 0 | Low ADNC | - | - | 1 | 1 | 1 |
| HNL12 | L | 68 | F | 0.67 | 0 | Intermediate ADNC | CAA | LATE | 3 | 2 | 3 |
| NDRI01 | L | 82 | M | 0.5 | 0 | - | - | - | - | - | - |

**Supplementary Table 2: Subject demographic and neuropathological information.** Neuropathological diagnosis data and ABC scores are not available for the NDRI specimen. The average pathology ratings are based on histological samples from the hemisphere ipsilateral to the thickness measures whereas the neuropathological diagnoses and ABC scores are based on histological samples taken from the contralateral hemisphere. For the ex vivo thickness analysis, the average pathology rating across three MTL locations (anterior entorhinal cortex, dentate gyrus and cornu ammonis) was used as a measure of general pathology burden in a specimen. Ratings were provided on a scale of 0-3, where 0 means no/rare pathology and 3 means severe pathology. (ADNC = Alzheimer’s disease neuropathologic change; CBD = Corticobasal Degeneration; FTLD-TDP= Frontotemporal Dementia (with TDP-43 inclusions); LBD = Lewy Body Disease; CVD = Cerebrovascular Disease; PSP = Progressive Supranuclear Palsy; PART = Primary age-related tauopathy; CAA = Cerebral amyloid angiopathy; LATE = Limbic-predominant age-related TDP-43 encephalopathy)


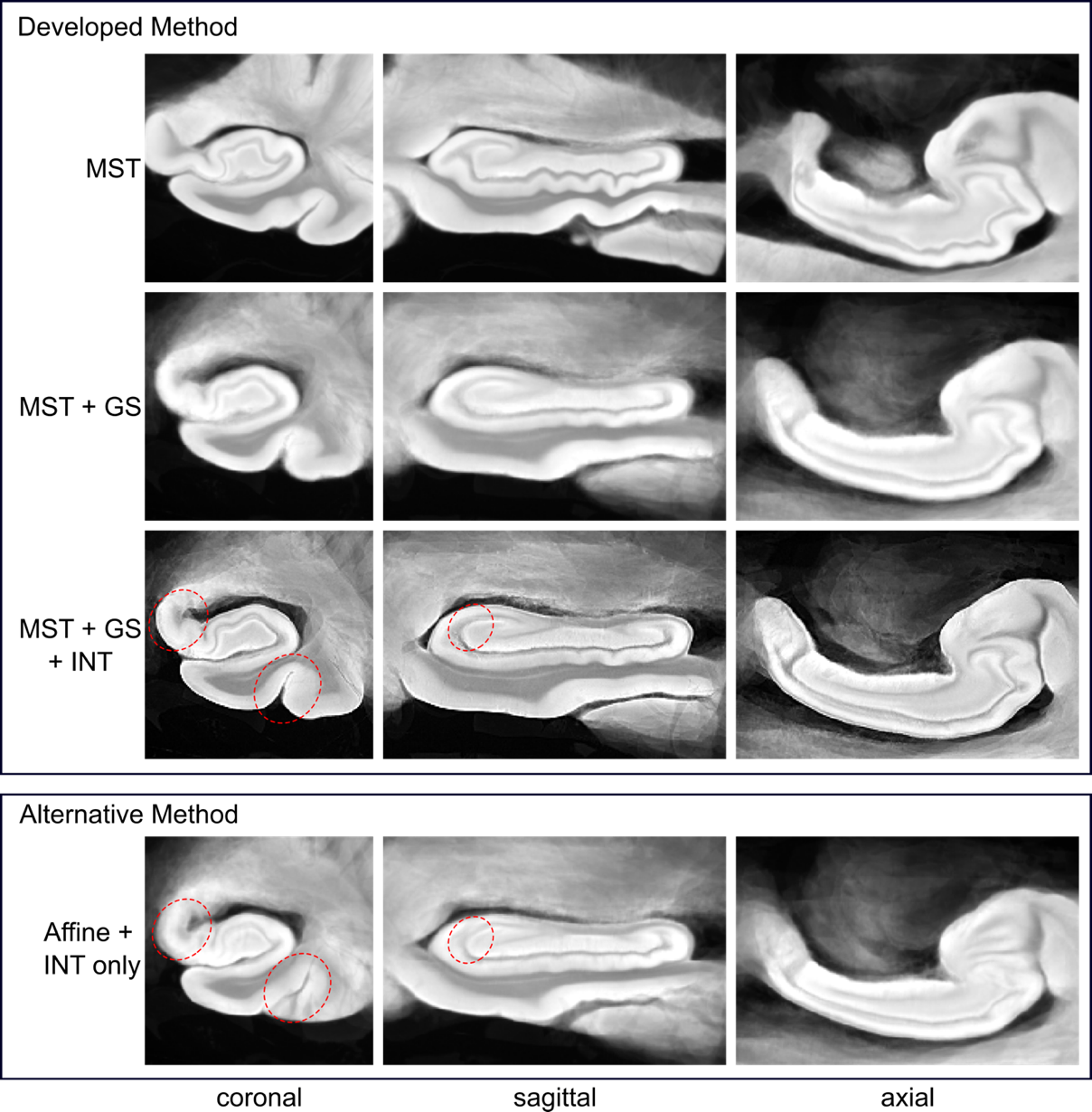


**S****upplementary Figure 2: Qualitative comparison of the MRI atlases generated by each stage of the atlas pipeline: minimum spanning tree (MST), geodesic shooting (MST + GS) and intensity-based groupwise registration (MST + GS + INT).** The bottom row shows the atlas obtained using an alternate approach: affine alignment of the segmentations, followed by intensity-based groupwise registration (INT only). The dashed red circles highlight areas where the developed atlas (MST + GS + INT) shows a visible improvement compared to the “INT only” atlas.


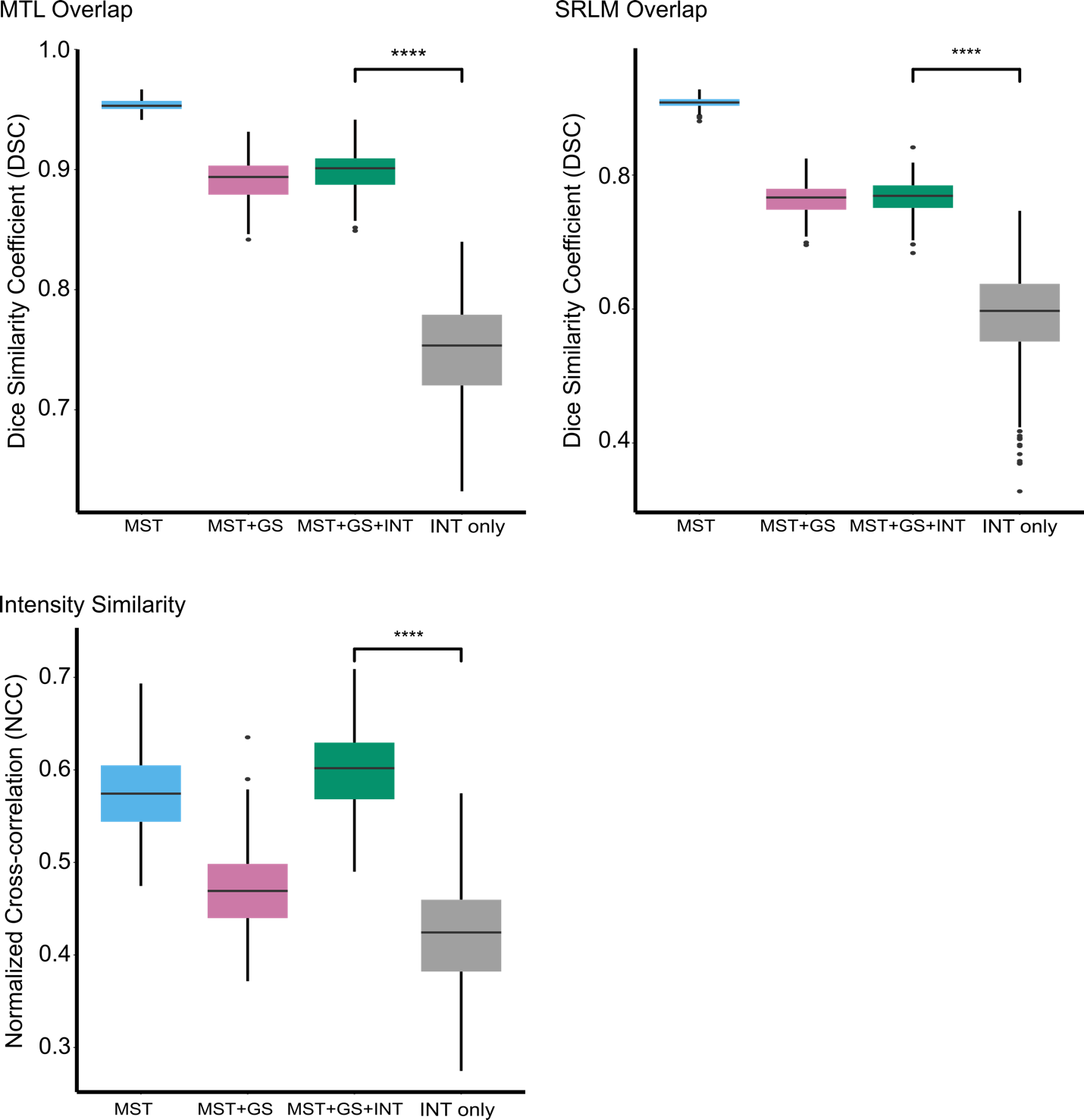


**Supplementary Figure 3: Quantitative metrics of the registration quality after each stage of the atlas construction pipeline, and the alternate “INT only” approach. Asterisks indicate statistically significant differences (****: p < 0.0001, paired t-test).** For the “MST” and “MST+GS” stages, the Dice Similarity Coefficient (DSC) coefficient is high, and the Normalized Cross-Correlation (NCC) is low since these stages perform registration using the segmentation images, rather than MRI itself. After the INT stage, the NCC increases (indicating better matching of MRI intensity), and the DSC remains relatively high. Without the “MST” and “MST+GS” steps, the affine followed by intensity-only registration (INT only) fails to converge to as good of a solution as “MST+GS+INT”. Thus, the “MST+GS” steps provide a better initialization for intensity-based registration compared to groupwise affine registration. See text (Section S2.1) for a detailed explanation.


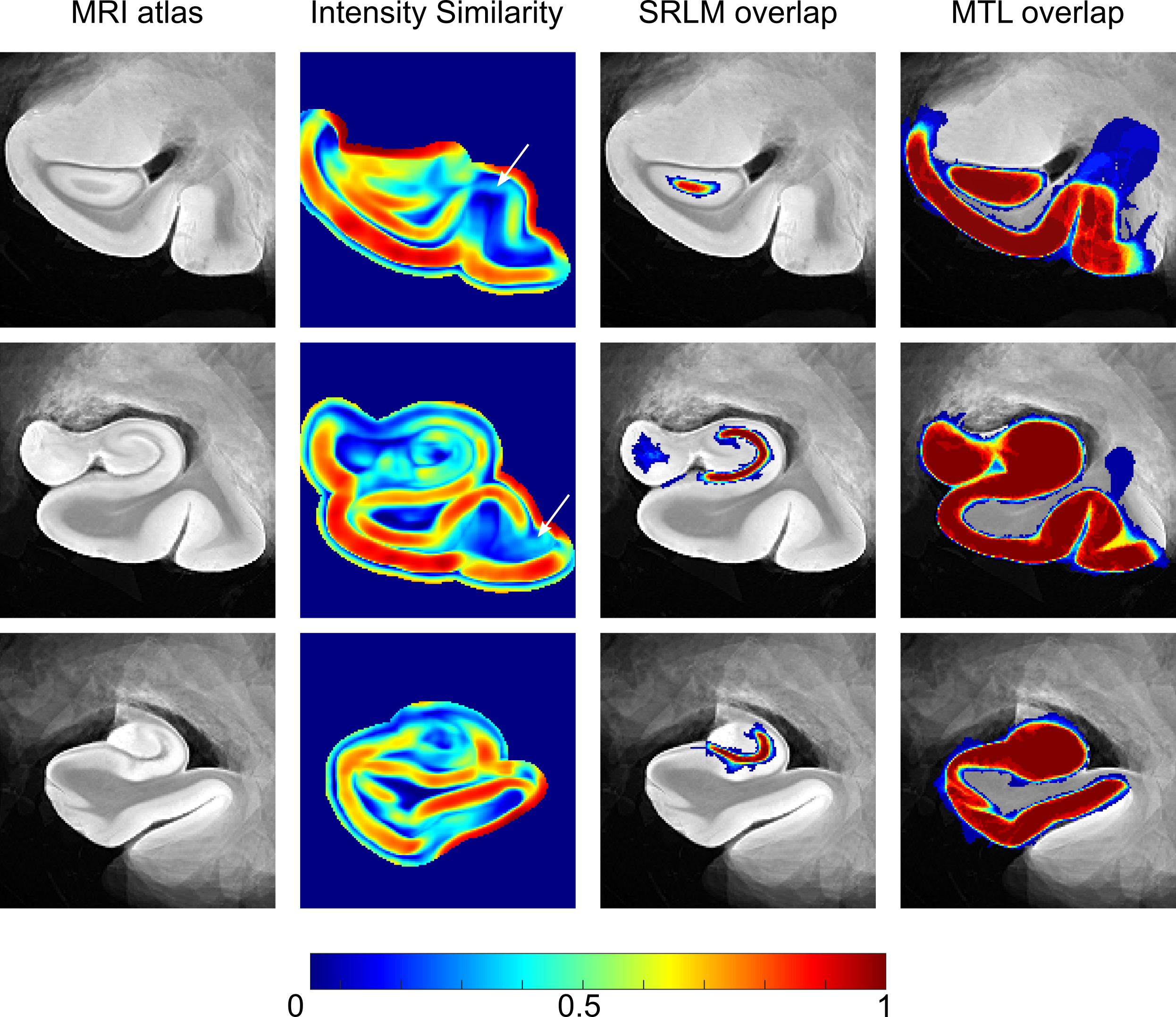


**Supplementary Figure 4*:*** Quantitative maps showing the registration quality, averaged across all specimens in the space of the final atlas. Each row shows a coronal cross-section through the medial temporal lobe, going from an anterior level (top) to a posterior level (bottom). The ‘Intensity Similarity’ images in the second column represent the average normalized cross-correlation (NCC) metric between each pair of specimens registered to the atlas. Columns three and four show the degree of overlap between the segmentations of all specimens registered to the atlas. The white arrows indicate regions surrounding the collateral sulcus in the anterior MTL where the registration quality is the poorest.

**
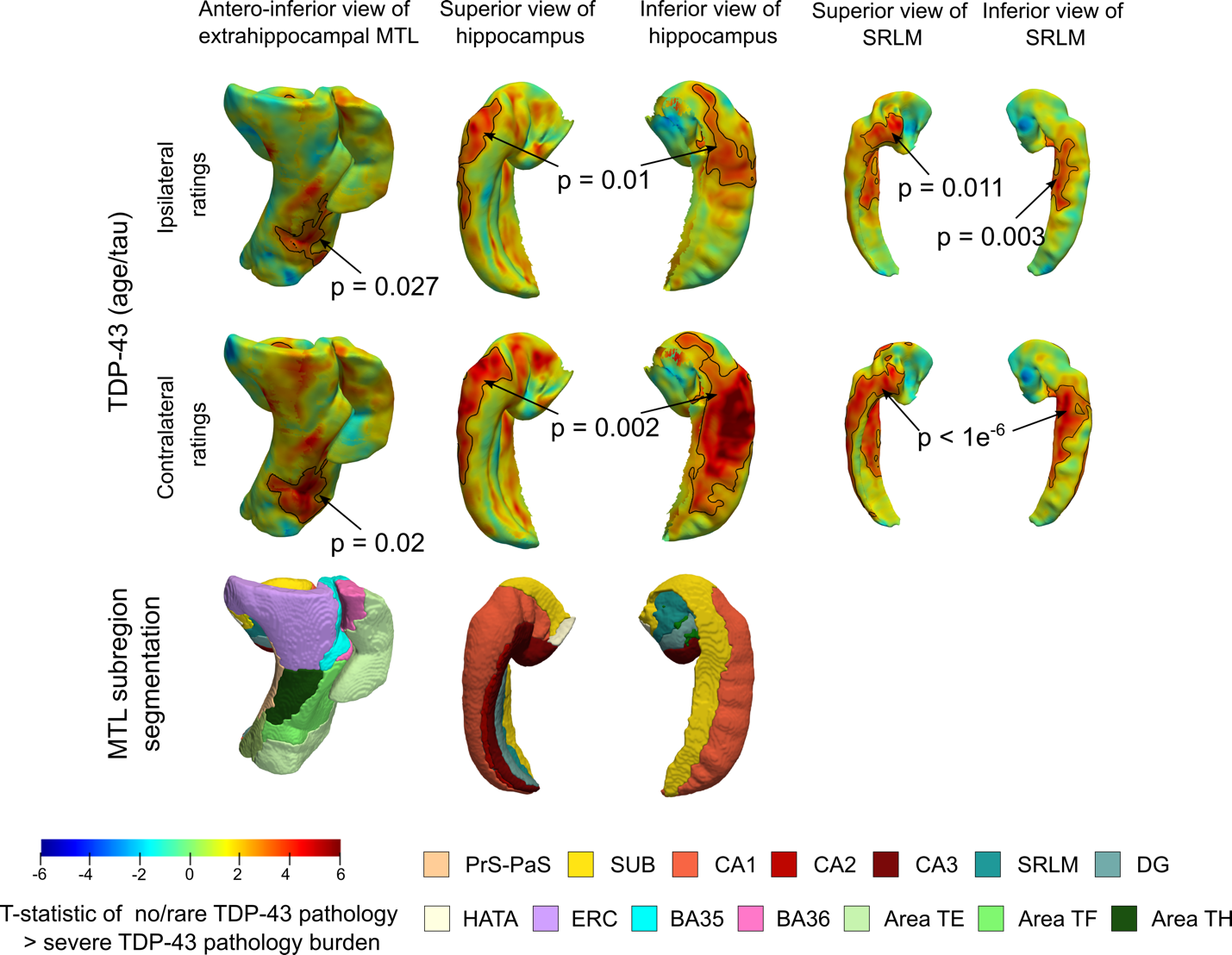
**

**Supplementary Figure 5: Statistical map of the correlation between cortical thickness and the severity of TDP-43 pathology using ratings derived from the hemisphere both ipsilateral (N = 27) and contralateral (N = 26) to the thickness measures.** These analyses were performed in the subset of specimens age 59 years and older. The covariates used in each analysis are provided in parentheses. The clusters outlined in black indicate regions where a significant correlation was observed after correction for multiple hypothesis testing (corrected p < 0.05). (SUB = subiculum; SRLM = stratum radiatum lacunosum moleculare; CA = cornu ammonis; DG = dentate gyrus; HATA = hippocampal amygdala transition area; ERC = entorhinal cortex; BA = Brodmann Area)

**
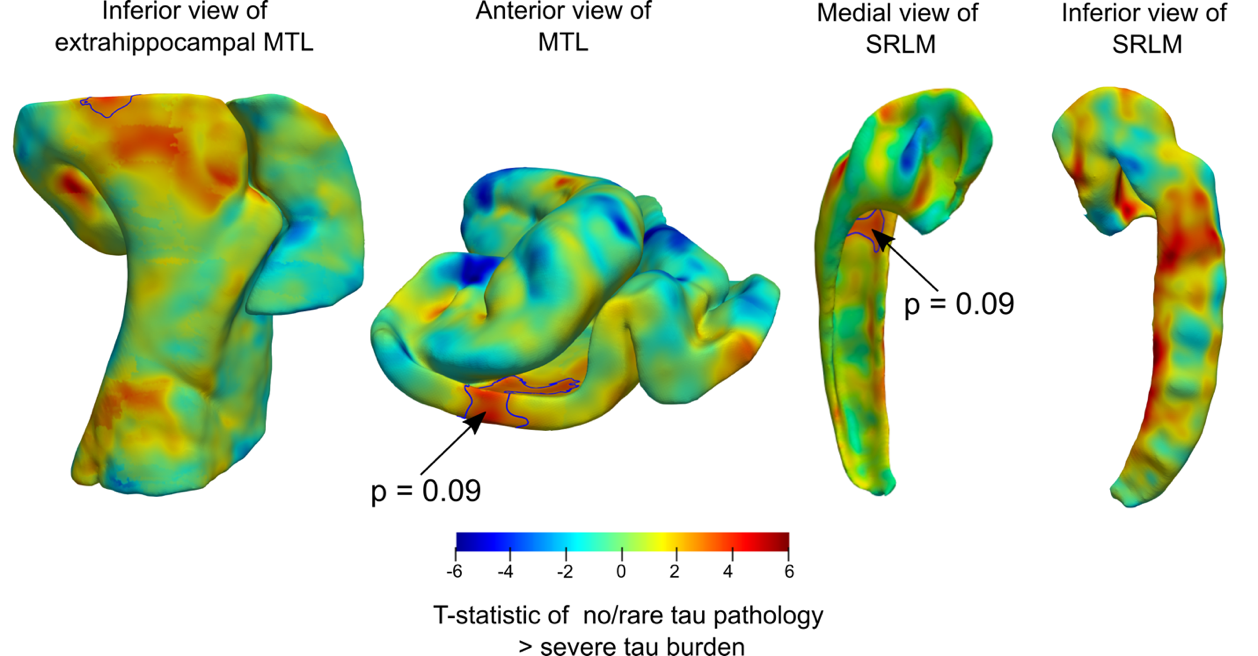
**

**Supplementary Figure 6**: **Statistical analysis correlating the severity of tau pathology and cortical thickness, with covariates for age and TDP-43 pathology, performed in the subset of specimens from donors age 59 and older after exclusion of case CNDR06, which is potentially affected by brain swelling (N = 26).** The clusters outlined in blue in the ERC (corrected p = 0.09) and SRLM (corrected p = 0.09) indicate regions where trend level correlations were observed after correction for multiple hypothesis testing.

**
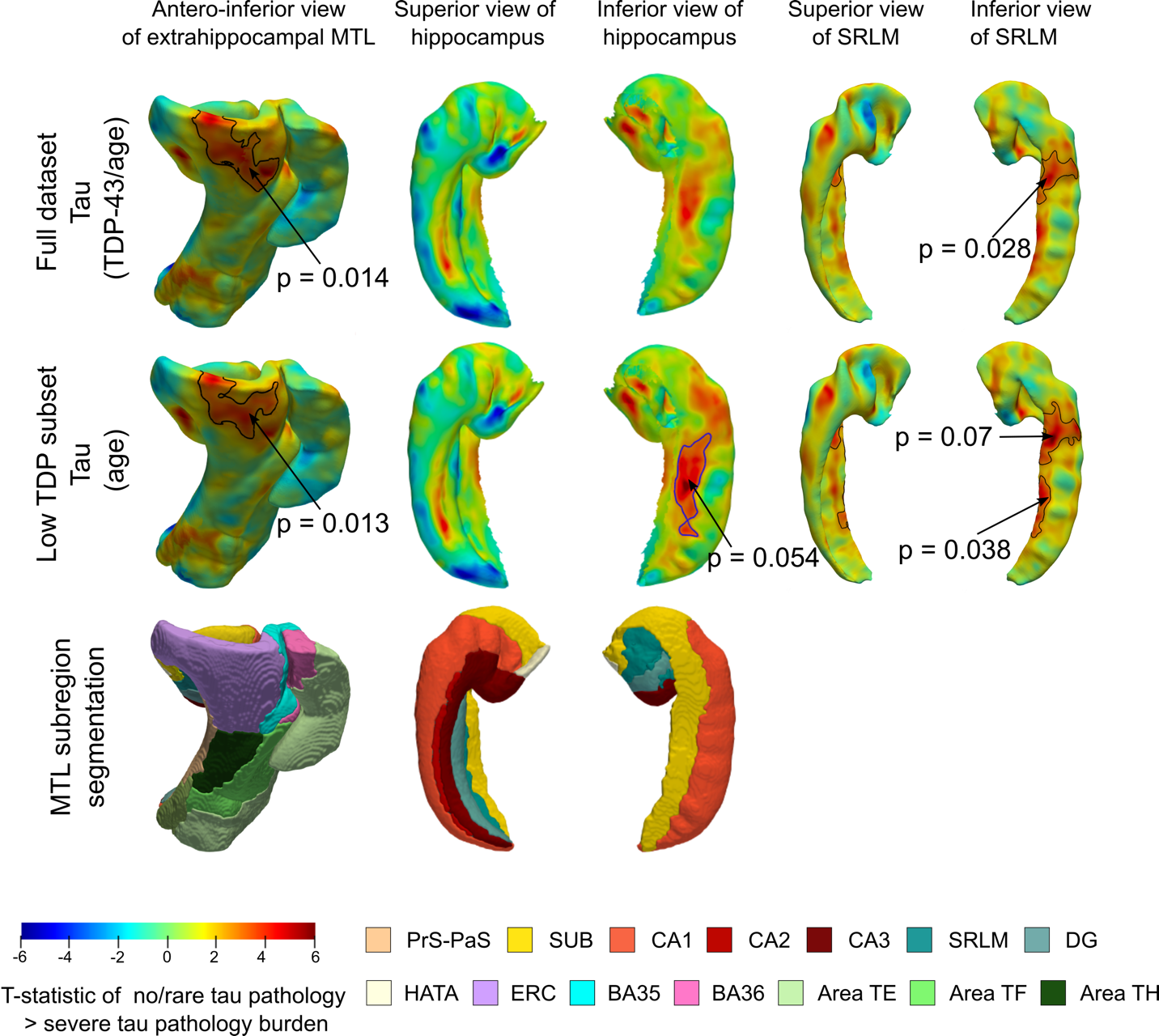
**

**Supplementary Figure 7: Statistical comparison of cortical thickness and the severity of tau pathology after calculating MTL thickness in the native space of each specimen instead of distortion corrected space.** The covariates used in each analysis are provided in parentheses. The clusters outlined in black indicate regions where a significant correlation was observed after correction for multiple hypothesis testing (corrected p < 0.05). The clusters outlined in blue indicate regions where a trend level correlation was observed (corrected p < 0.10). (SUB = subiculum; SRLM = stratum radiatum lacunosum moleculare; CA = cornu ammonis; DG = dentate gyrus; HATA = hippocampal amygdala transition area; ERC = entorhinal cortex; BA = Brodmann Area)


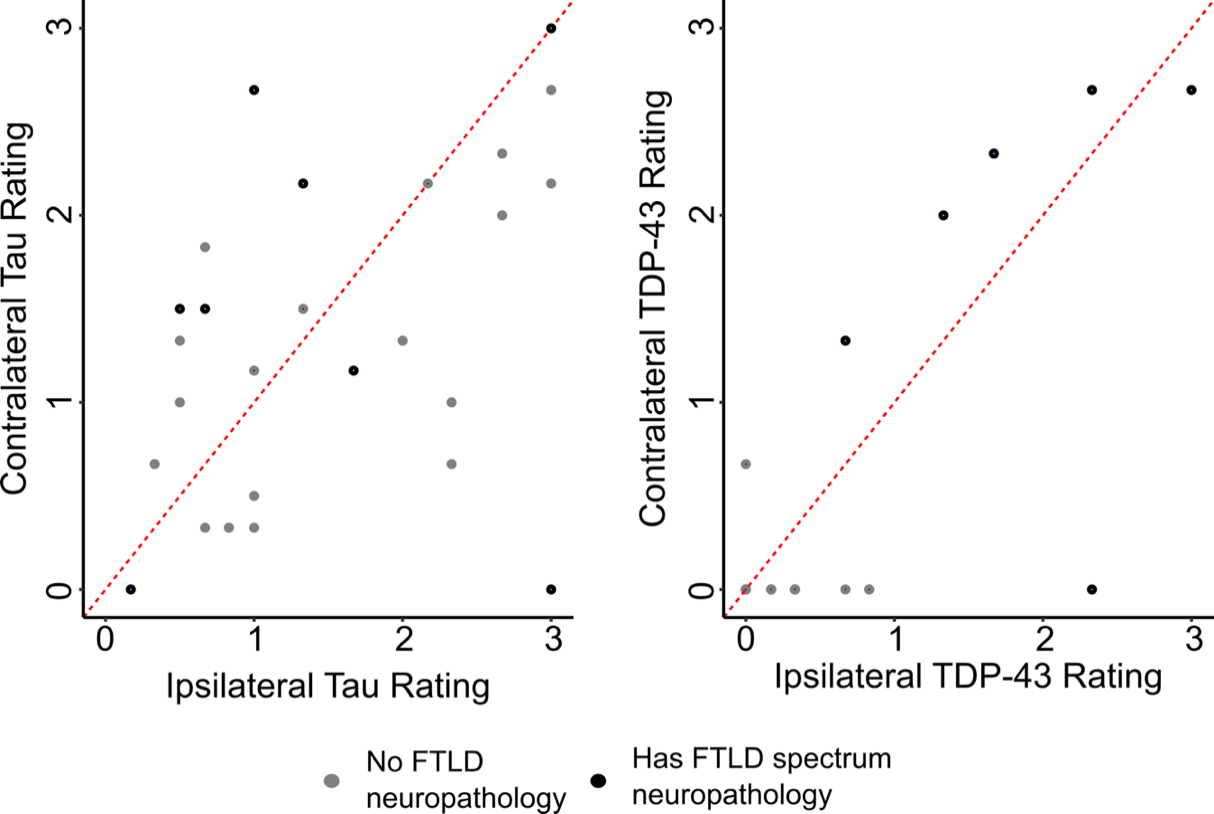


**Supplementary Figure 8**: **Scatterplots of the relationship between average ipsilateral and contralateral ratings of tau and TDP-43 pathology.** The dashed red line indicates the line of equality**.** A different color is used to indicate cases with frontotemporal lobar degeneration (FTLD) neuropathology.


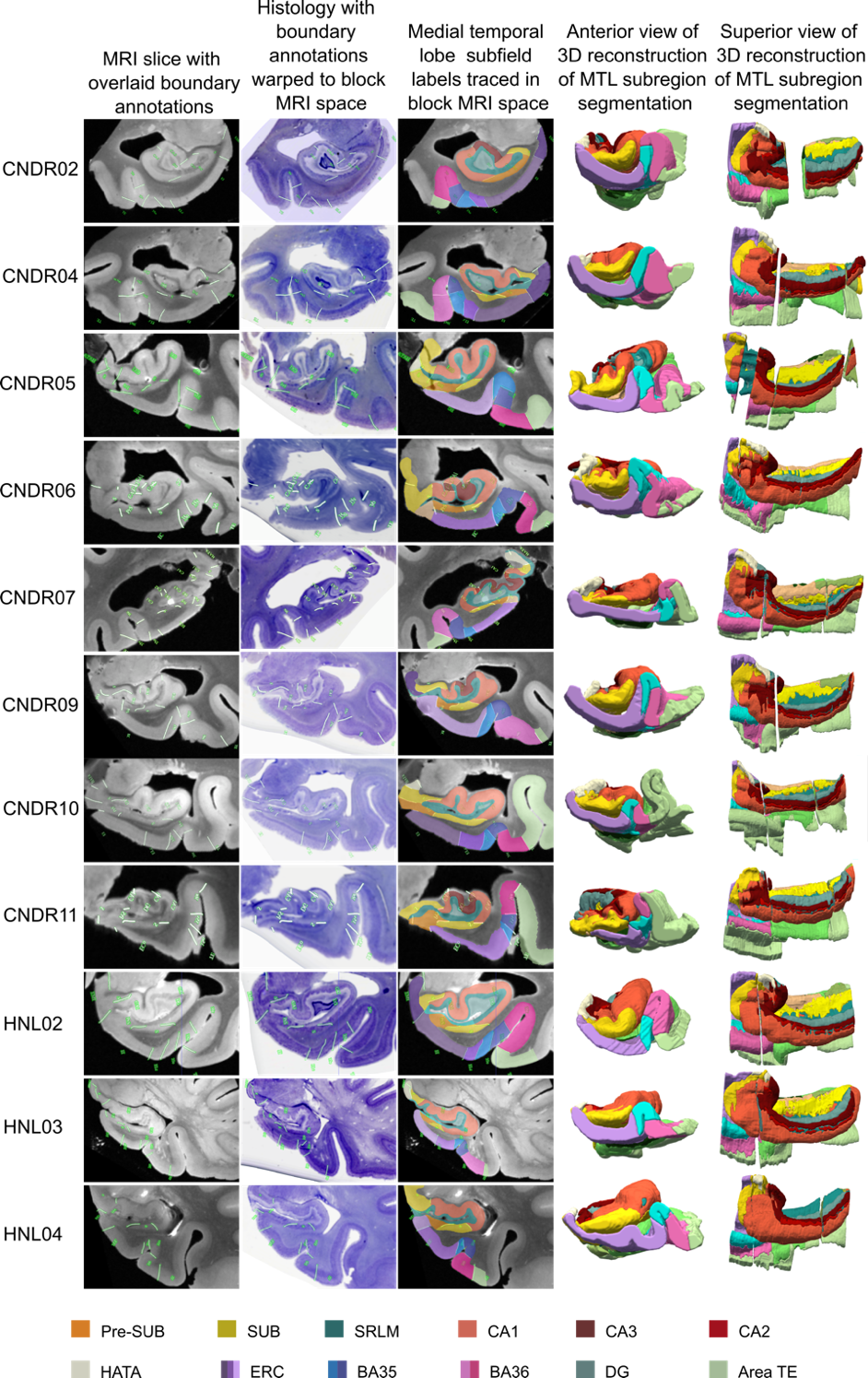


**Supplementary Figure 9: Example coronal cross-sections showing the histology annotations mapped into MRI space and used to guide the segmentation of the medial temporal lobe (MTL) subregions and 3D reconstructions of the completed MTL subregion segmentation.** The white markings on each image are annotations of subregion boundaries, identified on the basis of cytoarchitectural features in the histology image. For each of the eleven specimens, an anterior cross-sectional visualization is provided of corresponding slices of the MRI scan, digital histology image and cytoarchitecture-guided MTL subregion segmentation, as well as two views of the 3D reconstructed segmentation. Small gaps in the reconstructed segmentation exist between histology blocks. Note that fine-grained segmentation labels provided for the subregions of ERC, BA35 and BA36 have been grouped together in the figure legend. This figure also illustrates the variability in the folding of the collateral sulcus in different specimens and its influence on the location of BA35. (SUB = subiculum; SRLM = stratum radiatum lacunosum moleculare; CA = cornu ammonis; DG = dentate gyrus; HATA = hippocampal amygdala transition area; ERC = entorhinal cortex; BA = Brodmann Area)

**Supplementary Figure 10: Example images sampled from ipsilateral tau immunohistochemistry (IHC) sections at the location corresponding to each of the AD-specific “hotspots” for each specimen, visualized in order of decreasing median thickness within the hotspot.** This figure uses the hotspots shown in Fig. 4B. Sections are stained using AT8, a human phosphorylated tau antibody IHC stain. In 17 cases with serial tau IHC, the histology section closest to center of the hotspot is shown. For the remaining 12 cases, tau IHC was only performed on two sections for neuropathological diagnosis. In these cases, the location of the anterior entorhinal cortex hotspot matched most closely with the histology section taken at the mid-level of the amygdala, and the location of the subiculum and SRLM hotspots matched more closely with the section at the level of the hippocampal body. Cases outlined in red have a primary diagnosis with FTLD-Tau neuropathology or argyrophilic grain disease. More detailed clinical information for each specimen is provided in Supplementary Table 2. Larger histology images, with an expanded microscopic field and corresponding zoomed out views indicating the exact location where the MTL was sampled are provided for each specimen as supplementary material (<https://doi.org/10.6084/m9.figshare.15878913.v3>). We note that histology sections were sampled arbitrarily within the region of the hotspot by author S.R., and not specifically at locations where NFTs were visible. * For CNDR10 and 12, the tau IHC sections are shown at a larger scale in Supplementary Figure 11.

***
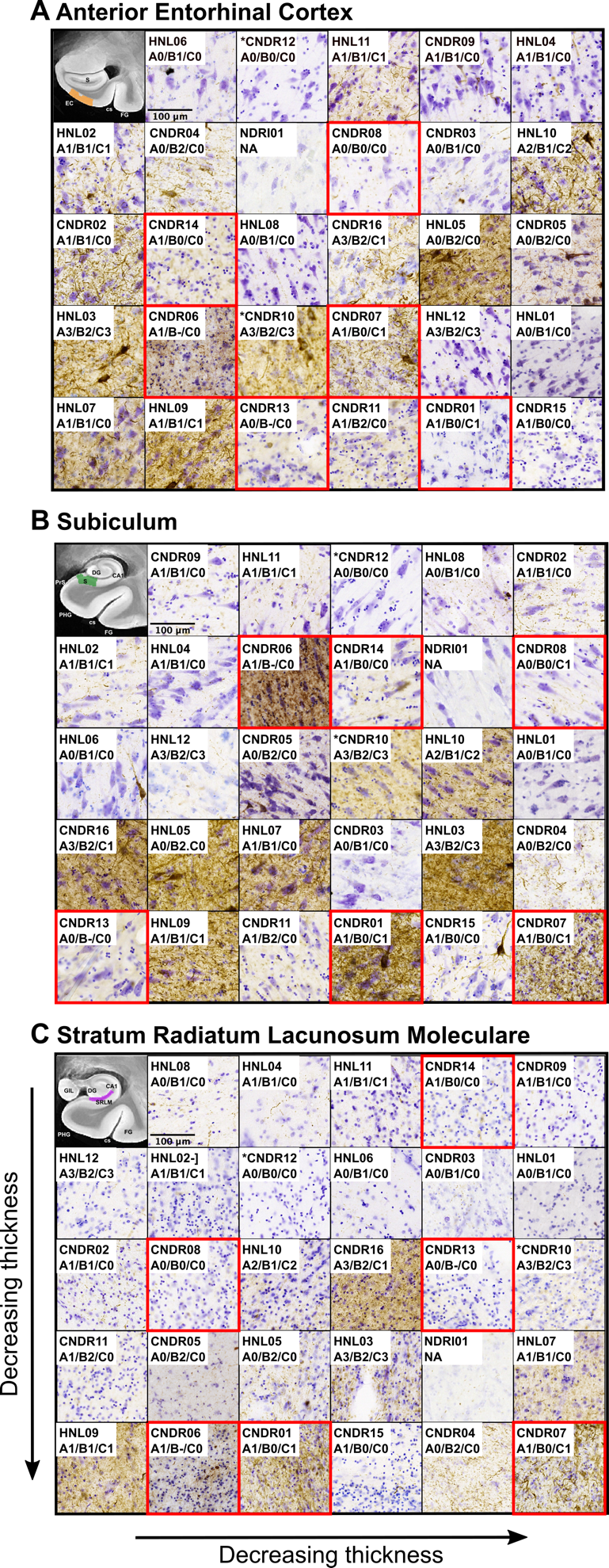
***

**
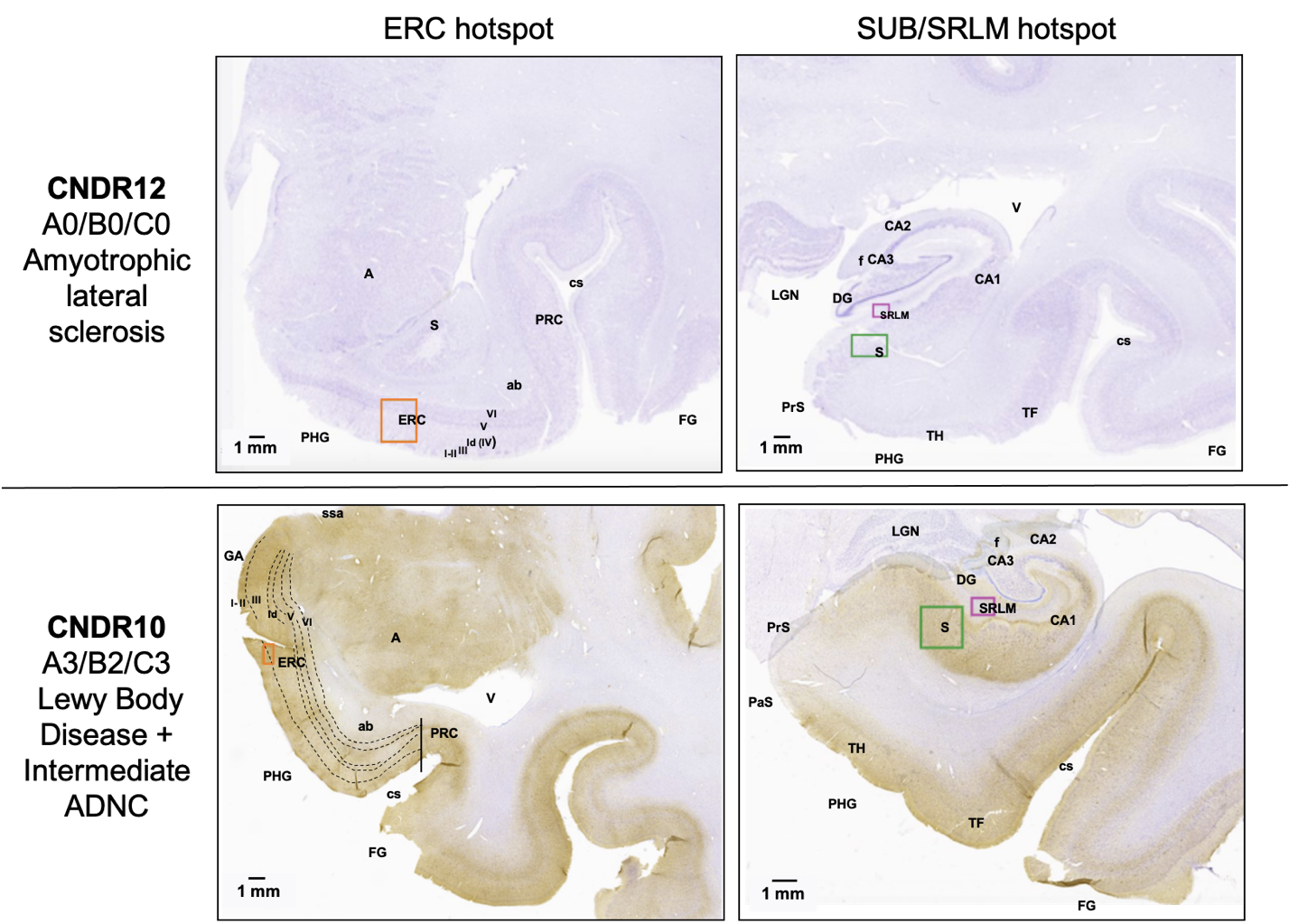
**

**Supplementary Figure 11: As a complement to Supplementary Fig. 10, this figure shows large-scale views of the tau immunohistochemistry (IHC) sections for two cases (CNDR10 and 12), taken at the levels corresponding to the each of the AD-specific “hotspots”.** The histology images have been marked to show the different layers of the cortex and the surrounding anatomical subregions. (A = amygdala, ab = angular bundle, CA = cornu ammonis, cs = collateral sulcus, DG = dentate gyrus, ERC= entorhinal cortex, FG = fusiform gyrus, f = fimbria, GA = gyrus ambiens, LGN = lateral geniculate nucleus, PHG = parahippocampal gyrus, PRC= perirhinal cortex, S = subiculum, Pr/Pa-S = pre/para-subiculum, SRLM = stratum radiatum lacunosum moleculare, ssa = sulcus semianularis, TH = Area TH, TF = Area TF, V = lateral ventricle, ADNC = Alzheimer’s disease neuropathologic change)

***
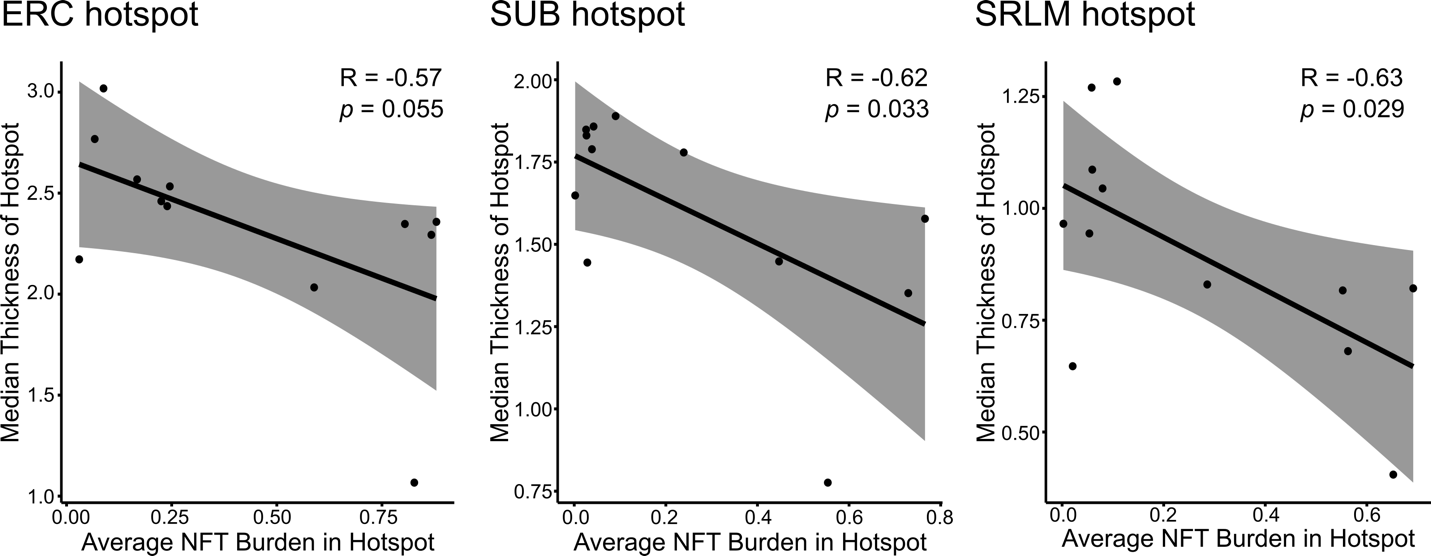
***

**Supplementary Figure 12: Scatter plots showing the correlation between the average NFT burden computed within each atrophic hotspot (using quantitative data from Yushkevich et al.** [14]**) and the median thickness of each hotspot (N = 12).** Cases with primary FTLD-Tau and argyrophilic grain disease were excluded from this analysis. The hotspots used in this analysis correspond to the result shown in Fig. 4B. (ERC = entorhinal cortex; SUB = subiculum; SRLM = stratum radiatum lacunosum moleculare). No covariates are included in these analyses because of the small sample size.

**Supplementary References**

1. Adler DH, Wisse LEM, Ittyerah R, Pluta JB, Ding SL, Xie L, Wang J, Kadivar S, Robinson JL, Schuck T, Trojanowski JQ, Grossman M, Detre JA, Elliott MA, Toledo JB, Liu W, Pickup S, Miller MI, Das SR, Wolk DA, Yushkevich PA (2018) Characterizing the human hippocampus in aging and Alzheimer’s disease using a computational atlas derived from ex vivo MRI and histology. Proc Natl Acad Sci U S A 115:4252–4257. doi: 10.1073/pnas.1801093115

2. Allassonnière S, Trouvé A, Younes L (2005) Geodesic Shooting and Diffeomorphic Matching Via Textured Meshes. Energy Minimization Methods Comput Vis Pattern Recognit - EMMCVPR 2005 3757:365–381

3. Corsini M, Cignoni P, Scopigno R (2012) Efficient and Flexible Sampling with Blue Noise Properties of Triangular Meshes. IEEE Trans Vis Comput Graph 18:914–924. doi: 10.1109/TVCG.2012.34

4. Crum WR, Camara O, Hill DLG (2006) Generalized Overlap Measures for Evaluation and Validation in Medical Image Analysis. IEEE Trans Med Imaging 25:1451–1461. doi: 10.1109/TMI.2006.880587

5. Dou Q, Chen H, Jin Y, Yu L, Qin J, Heng P-A 3D Deeply Supervised Network for Automatic Liver Segmentation from CT Volumes

6. Joshi S, Davis B, Jomier M, Gerig G (2004) Unbiased diffeomorphic atlas construction for computational anatomy. Neuroimage 23. doi: 10.1016/j.neuroimage.2004.07.068

7. Lorensen WE, Cline HE (1987) Marching cubes: A high resolution 3D surface construction algorithm. In: Proceedings of the 14th Annual Conference on Computer Graphics and Interactive Techniques, SIGGRAPH 1987. Association for Computing Machinery, Inc, pp 163–169

8. Ogniewicz RL, Kübler O (1995) Hierarchic Voronoi skeletons. Pattern Recognit 28:343–359. doi: 10.1016/0031-3203(94)00105-U

9. Ravikumar S, Wisse L, Gao Y, Gerig G, Yushkevich P (2019) Facilitating Manual Segmentation of 3D Datasets using Contour and Intensity Guided Interpolation. In: 2019 IEEE 16th International Symposium on Biomedical Imaging (ISBI 2019). pp 714–718

10. Ronneberger O, Fischer P, Brox T (2015) U-net: Convolutional networks for biomedical image segmentation. In: Lecture Notes in Computer Science (including subseries Lecture Notes in Artificial Intelligence and Lecture Notes in Bioinformatics). Springer Verlag, pp 234–241

11. Tustison NJ, Avants BB, Cook PA, Yuanjie Zheng Y, Egan A, Yushkevich PA, Gee JC (2010) N4ITK: Improved N3 Bias Correction. IEEE Trans Med Imaging 29:1310–1320. doi: 10.1109/TMI.2010.2046908

12. Vaillant M, Miller MI, Younes L, Trouvé A (2004) Statistics on diffeomorphisms via tangent space representations. Neuroimage 23:161–169. doi: 10.1016/j.neuroimage.2004.07.023

13. Winkler AM, Ridgway GR, Webster MA, Smith SM, Nichols TE (2014) Permutation inference for the general linear model. Neuroimage 92:381–397. doi: 10.1016/j.neuroimage.2014.01.060

14. Yushkevich PA, López MM, Martin MMI de O, Ittyerah R, Lim S, Ravikumar S, Bedard ML, Pickup S, Liu W, Wang J, Hung LY, Lasserve J, Vergnet N, Xie L, Dong M, Cui S, McCollum L, Robinson JL, Schuck T, de Flores R, Grossman M, Tisdall MD, Prabhakaran K, Mizsei G, Das SR, Artacho-Pérula E, Jiménez M del MA, Rabal MPM, Romero FJM, Sánchez SC, González JCD, de la Rosa-Prieto C, Parada MC, Lee EB, Trojanowski JQ, Ohm DT, Wisse LEM, Wolk DA, Irwin DJ, Insausti R (2021) Three-dimensional mapping of neurofibrillary tangle burden in the human medial temporal lobe. Brain 139:16–17. doi: 10.1093/BRAIN/AWAB262

15. Yushkevich PA, Piven J, Hazlett HC, Smith RG, Ho S, Gee JC, Gerig G (2006) User-guided 3D active contour segmentation of anatomical structures: Significantly improved efficiency and reliability. Neuroimage 31:1116–1128. doi: 10.1016/j.neuroimage.2006.01.015

16. Zeng G, Yang X, Li J, Yu L, Heng PA, Zheng G (2017) 3D U-net with multi-level deep supervision: Fully automatic segmentation of proximal femur in 3D MR images. In: Lecture Notes in Computer Science (including subseries Lecture Notes in Artificial Intelligence and Lecture Notes in Bioinformatics). Springer Verlag, pp 274–282

1. Prior to 2017, specimens were imaged using a narrower 35mm coil as described in Adler et al. (Specimens: CNDR01-04, NDRI02) [↑](#footnote-ref-1)
